# Individual Genetic Heterogeneity

**DOI:** 10.3390/genes13091626

**Published:** 2022-09-10

**Authors:** Mauno Vihinen

**Affiliations:** Department of Experimental Medical Science, BMC B13, Lund University, SE-22184 Lund, Sweden; mauno.vihinen@med.lu.se

**Keywords:** genetic heterogeneity, genetic variation, somatic variation, TARAR countermeasures, epigenetics, selfish genetic element

## Abstract

Genetic variation has been widely covered in literature, however, not from the perspective of an individual in any species. Here, a synthesis of genetic concepts and variations relevant for individual genetic constitution is provided. All the different levels of genetic information and variation are covered, ranging from whether an organism is unmixed or hybrid, has variations in genome, chromosomes, and more locally in DNA regions, to epigenetic variants or alterations in selfish genetic elements. Genetic constitution and heterogeneity of microbiota are highly relevant for health and wellbeing of an individual. Mutation rates vary widely for variation types, e.g., due to the sequence context. Genetic information guides numerous aspects in organisms. Types of inheritance, whether Mendelian or non-Mendelian, zygosity, sexual reproduction, and sex determination are covered. Functions of DNA and functional effects of variations are introduced, along with mechanism that reduce and modulate functional effects, including TARAR countermeasures and intraindividual genetic conflict. TARAR countermeasures for tolerance, avoidance, repair, attenuation, and resistance are essential for life, integrity of genetic information, and gene expression. The genetic composition, effects of variations, and their expression are considered also in diseases and personalized medicine. The text synthesizes knowledge and insight on individual genetic heterogeneity and organizes and systematizes the central concepts.

## 1. Introduction

Genetic variation is pervasive, all individuals within a species differ somewhat in their genomes, and the genomes of individual cells in a multicellular organism contain differences. As an example, the genomes of humans are 99.9% identical [1]. This means that there are some 3 million single nucleotide variations (SNVs) per haploid genome (about 1 variant per 1000 bp [2]), and numerous other types of alterations in comparison to the reference genome. Genome in an organism is dynamic, somatic alterations are numerous, and accumulate during lifetime. The genomic constitution of each case of cancer is unique [3] and the number of variants in a cancer patient can exceed 1 million [4].

Variations are generated by several mechanisms. Evolution of sequences and species is based on genetic variation. Many variants are tolerated and do not have a phenotype, effect on fitness, or other consequences, whereas harmful variants cause genetic diseases. Beneficial variants can be enriched by selection during evolution.

Genetic variation is one form of pervasive and normal biological heterogeneity called poikilosis [5]. There is a balance between variation generation and repair. In a normal situation, efficient DNA repair systems correct almost all lesions; however, it would be extremely costly to detect and correct all changes, thus somatic variations accumulate during the lifespan. Several mechanisms reduce and attenuate effects of variations. The last option for cells with excessive or very severe variation(s) is programmed cell death.

The goal of this survey is to provide a comprehensive view on individual genetic heterogeneity, its origin, implementation of genetic information, functional and phenotypic effects, and constraints of functional effects. Population effects and phenomena are excluded.

Genetic variations can be described and grouped in many ways depending on the perspective and type of variation: type of nucleic acid; size or effect of variation; disease relevance; affected molecular level; variation generating mechanism; frequency; harmfulness (pathogenic/neutral/unknown); neutral, risk, and protective alleles; type of inheritance; chromosome type (sex chromosomes vs. autosomes); nuclear and organellar genetic variation; origin of variation (inherited/*de novo*/somatic/epigenetic); location in coding or noncoding DNA; conflict between different types of genetic elements and variations in them; etc.

Genetic information and material are in cells in the form of large oligonucleotides, DNA or RNA chains, as genes, pseudogenes, and other expressed segments. Variation types include those in hybrids, genome, chromosomes and more locally in DNA regions, epigenetic variants, and selfish genetic elements. All higher organisms form an ecosystem with microorganisms called holobiont. The genetic constitution and heterogeneity of microbiota is of great importance in health and in many diseases. Mutation rates vary widely for variation types that are dependent, e.g., on the sequence context. Somatic variation is ubiquitous in multicellular organisms. Related to the implementation of genetic information in organisms, types of inheritance, both monogenic and non-Mendelian inheritance, zygosity, sexual reproduction, and sex determination are covered. Functions of nucleic acid polymers and functional effects of variations are introduced, along with constraints of functional effects including TARAR countermeasures, individual genetic conflicts, and effects of microbiota. TARAR countermeasures affect the phenotype by processes in tolerance, avoidance, repair, attenuation, and resistance. Intraindividual genetic conflict adds further layers to phenotypic expression. Thus, the genetic composition, effects of variations, and their expression form a very complex system that is unique for each individual. Here, the topics mentioned above are also considered from the perspective of diseases.

Variations at population level are excluded. Population level concepts, such as mode of reproduction, types of fertilization, pregnant sex, type of breeder, mate choice, mating systems, and interindividual genetic conflicts are not covered here. Mechanisms that produce variations are also excluded apart from some notes, for recent reviews see [6,7]. The goal of the presentation is to synthesize knowledge and insight in individual genetic heterogeneity and to organize and systematize the central concepts.

## 2. Genes and Genome

Genetic material is in most organisms in the form of long DNA strands. Exceptions are some viruses which have RNA genome [8]. RNA viruses can be classified as those that possess double-stranded genome, and those with either positive or negative-sense single-stranded RNA genomes. There has been a long debate about RNA world and whether RNA or DNA was the original genetic material in the first living organisms [9]. Genomes contain genes that code for gene products and other regions, which may function, e.g., in regulation, for more detailed discussion see 9. Functional effects of DNA variations.

Genomes of individuals in a species differ in many places. The length of the haploid human XY reference genome is 3,117,275,501 bp (T2T-CHM13v2.0 https://www.ncbi.nlm.nih.gov/assembly/GCF_009914755.1/#/st, accessed on 10 August 2022). The full complement of genes in a clade is called the pangenome. Individuals share the core genome but have variable numbers of dispensable genes. The size of the dispensable genome varies, for example, in soybean it accounts 20% and in rice 43% of the size [10]. The products of many dispensable genes respond to stresses and various stimuli and are depleted in several cultivated barley strains [10]. The human African pangenome is about 10% larger than the reference genome [11]. The other human pangenomes for several populations include smaller amounts of novel sequences, reviewed in [12]. A total of 315 of the African insertions are within protein-coding genes and can lead to alternative forms of gene products [11].

The concept of a gene is instrumental for genetics and genetic variations. Many definitions have been provided, see, e.g., [13,14,15] and references therein; however, none of them describes the current concept of gene. Here, *gene is defined as (i) a continuous stretch of polynucleotide sequence, (ii) consecutive segments, or (iii) connected segments joined together to code for a functional gene product or products.* Essential for the gene function is that the nucleic acid sequence is transcribed. Gene products are either RNA or protein molecules.

The definitions and systematics described here form part of the Variation Ontology (VariO) that is dedicated for biological variation types, functions, properties, and structural details [16]. VariO is an evolving ontology used, e.g., in UniProtKB/SwissProt [17]. While systematizing the language, it has been necessary to introduce a small number of new concepts as terms in the ontology have to be unambiguous.

The systematics for functions of RNA and protein molecules have been described in VariO. The RNA functions are amino acid transfer, catalytic activity, regulation, information transfer, splicing function, and translation. Protein functions comprise catalysis, information transfer, movement, recognition, storage, structural functions, and transport. Together, these functions form the basis for life and all cellular and organismal events, reactions, and responses. The functions of DNA are described below.

Genetic material is either DNA or RNA. A single gene can code several gene products, which further can be modified in various ways. Proteoforms are protein forms coded by a single gene [18]. Numerous mechanisms can generate different forms of the encoded protein, comprising genetic variants (substitutions, deletions, insertions, indels), transcription and translation errors, mRNA processing (alternative splicing, alternative start and stop codons, (programmed) ribosomal frameshifting), post translational modifications such as proteolytic processing and amino acid modifications, as well as protein splicing [19]. A genetic region may contain several overlapping genes. Genes in prokaryotes and certain genes in eukaryotes are intronless and thus continuous. Introns divide genes into segments in eukaryotes. Genes for immunological recognition molecules, immunoglobulins, and B and T cell receptors are joined in VDJ recombination from independent segments [20]. The location of a gene does not have to be fixed. Transposable elements can move within the genome and code for gene products.

The gene-coding portions of genomes vary largely. Many viruses have so compact genomes that some genes overlap, usually in different reading frames. Overlap appears also in organisms with intergenic material; however, this is quite rare. In humans, the exons of protein coding genes account 2.94% and protein coding exons cover 1.22% of the genome [21]. Genes can be continuous or be split into exons and introns. When introns, promoters, and poly(A) sites are also included, the protein coding genes cover 39.54% of human genome [21]. Despite the small portion of coding region of genes, the entire or almost entire genome is transcribed [22]. ENCODE project has charted the human noncoding regions [23], functions of which are still largely unknown.

Pseudogenes are copies of genes that are not functional or not at least in the same way as the original gene. They have been identified in all types of organisms ranging from bacteria to mammals. The definition in VariO is *nucleic acid segment that resembles a functional gene, but which is defective*. Pseudogenes are nonfunctional copies of genes. They originate with three mechanisms [24]. Duplicated pseudogenes are produced via tandem duplication or uneven crossing over and processed pseudogenes arise by retrotransposition, when an mRNA sequence or part of it is reverse transcribed to DNA and inserted into the genome [25]. The human genome contains > 14 700 duplicated or retroposed pseudogenes according to the GENCODE database Release version 41 (https://www.gencodegenes.org/human/stats.html, accessed on 10 August 2022). The third form of pseudogenes, unitary pseudogene, is extremely rare. It is based on losing gene function without a functional copy. Polymorphic pseudogenes are classified as pseudogenes in the reference genome for a species, but can be functional in some individuals [24].

A duplicate of a gene quickly accumulates variants that destroy its coding region, introduce stop codons, etc., since there is no evolutionary pressure to keep pseudogenes active. However, some pseudogenes are expressed, also in a tissue-specific manner [26]. Despite nonfunctional sequences, the accumulation of variations does not occur at random rate in some pseudogenes, and they display evolutionary selection. Pseudogenes have functions, e.g., as regulatory elements at DNA, RNA, and protein level; they provide a reservoir of genetic material for increased genetic diversity, and some pseudogenes are translated [24,25].

## 3. Types of Variations

A very large number of variation types has been described at different levels. VariO facilitates systematic description of variations, their types, effects, consequences, and mechanisms [16]. The ontology has been updated and it organizes systematically the concepts described in this text. VariO-based detailed descriptions of variation types have been presented previously for DNA [27], RNA [28], and protein variants [29] along with examples, often in relation to human diseases.

In addition to changes to DNA sequence, epigenetic variants that do not change the actual sequence are also common. A further layer of variation is introduced by selfish genetic elements that can multiply themselves. Furthermore, changes in microbiota, microorganisms found in and on all multicellular organisms, can be substantial and be related also to diseases. Here, a summary is presented to highlight the multiple types and effects of different kinds of variants.

### 3.1. Hybrid

Organisms reproduce sexually or asexually. Sexual reproduction, which is discussed below, is important for the genetic composition of individuals and for the generation of new genetic combinations. Hybrids originate in sexual reproduction by mating two organisms of different breeds, varieties, species, genera, or families. Hybrids are rather common, although many species have reproductive barriers that prevent hybrid offspring production by genetic, physiological, and behavioral processes.

Many hybrids are sterile [30], e.g., because of different number of chromosomes in comparison to parent species. In a hybrid, egg and sperm cells of different species are combined, therefore all the cells contain a mixture of genomes from both parental species. Intra-specific hybrids are produced by different subspecies of a species. Interspecific hybrids are hybrids between two different species within the same genus. Human (*Homo sapiens)* and Neanderthal hybrids were either intraspecific or interspecific. There has been a long debate whether Neanderthals were a separate species or a subspecies (*Homo neanderthalensis* or *Homo sapiens neanderthalensis*).

Intergeneric hybrid combines two genera within a family. Genera that produce intergeneric hybrids are always genetically related members of the same taxonomic tribe. An example is hybrid between gelada (*Theropithecus gelada*) and common baboon (*Papio hamadryas*), which interbreed occasionally in the wild [31]. Interfamilial hybrids are very rare; however, some instances are known, e.g., in kelps, brown algae seaweeds [32]. Hybridization has been used for development of novel species, especially in plant breeding to provide genetic variation and to improve properties [33].

### 3.2. Genome Wide Variations

**Figure 1:**
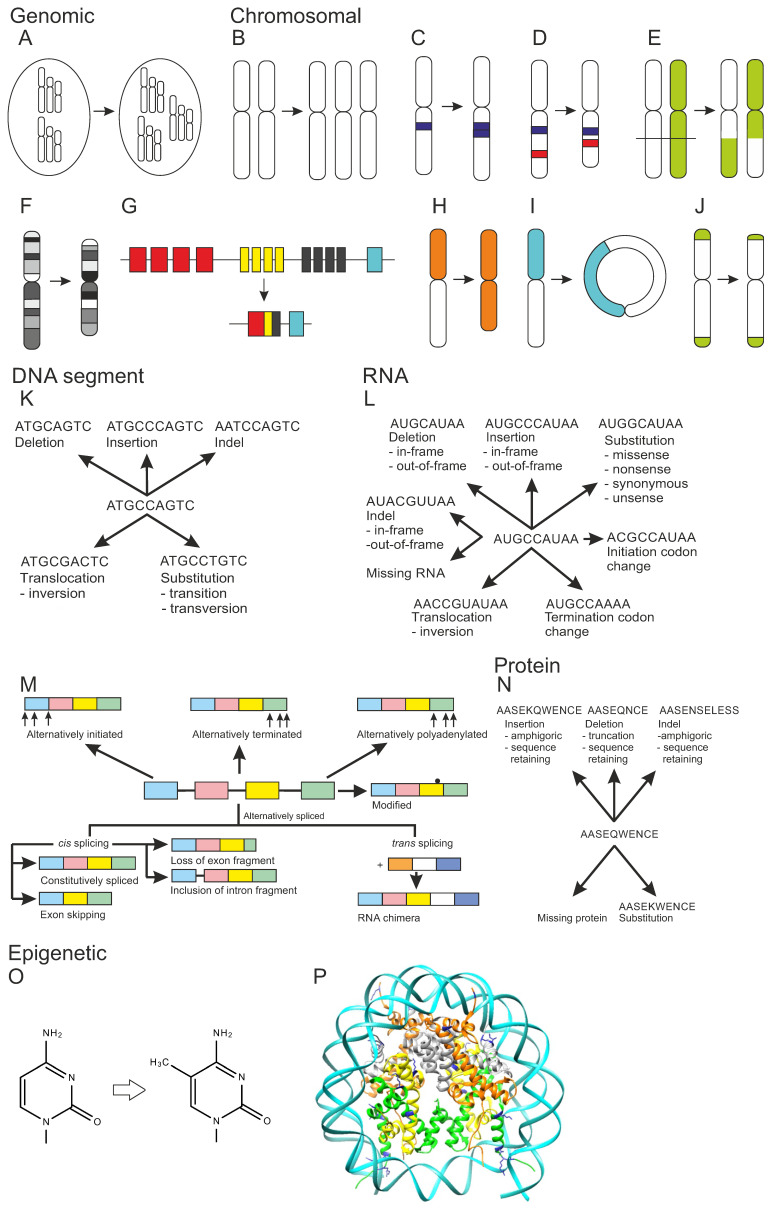
Types of genetic variations. Genomic variation: (**A**) Euploidy, altered number of genome sets. Chromosomal variation: (**B**) Aneuploidy, chromosome number variation, trisomy as an example. (**C**) Chromosomal amplification, (**D**) interstitial chromosomal deletion, (**E**) chromosomal translocation of type reciprocal translocation, (**F**) complex chromosomal variation, (**G**) immunological recognition molecule diversification by immunological receptor gene rearrangement, (**H**) isochromosome, (**I**) ring chromosome, and (**J**) telomere length variation. Types of DNA segment variation: (**K**) DNA variation types. RNA variation: (**L**) types of RNA variations, (**M**) mRNA forms. Protein variation: (**N**) types of protein variations. Epigenetic variation: (**O**) DNA methylation, cytosine methylation at C5 as example, (**P**) histone modification. Three-dimensional structure of nucleosome, DNA wound around histones (PDB code 6pwv [37]). DNA is in cyan, histones H2A orange, H2B gray, H3 green, H4 yellow. Post translational modification sites in blue, the majority of these sites are not shown as the flexible termini are not included to the structure. Adapted from [27,28,29].

Euploidy means change in the number of complete genome copies. Normal human and animal genomes are diploid, i.e., contain two copies of all chromosomes (Figure 1A). Genome duplications are rather common; however, much higher forms of ploidy have been detected. Many organisms, including humans, have underwent genome duplication during evolution [34]. The resulting duplicated genes are called ohnologs.

Anuclear cells lacking DNA, such as erythrocytes and platelets, are the most common in the human body comprising about 90% of the total number of cells [35]. These cells do not contain a nucleus and are lacking DNA. These cell types are highly differentiated and do not need DNA for their function, for example, erythrocytes are specialized oxygen transporters. Higher ploidies in humans appear in specialized cells, such as in the liver, heart, bone marrow, and placenta. In human liver, about 50% of cells have more than two sets of chromosomes [36].

In plants, endopolyploidy levels up to 24,576C have been identified [38]. 1C means haploid genome. In insects, polyploidy ranges from 4C to 2048C, the latter detected in salivary glands in *Drosophila* [39]. Polyploidy has been linked to high metabolic or synthetic activity, compensation for lack of nuclear DNA, response to environmental conditions, and to relationship between cell volume and DNA content [38]. Polyploidy reversal can reduce the degree of polyploidy by reductive mitosis, nuclear budding, or nucleophagy [40].

There are two forms of polyploidy. In autopolyploidy, the chromosomes are from one taxon [41], whereas in allopolyploidy they originate at least from two taxa. Mixoploid means an organism with different numbers of genome sets in different cells. This is very common in multicellular organisms.

Autoploidy and allopolyploidy can be further divided into categories [42]. Strict autopolyploidy and interracial autopolyploidy are the two forms of autopolyploidy. Allopolyploidy can be subdivided as segmental, true, also called genomic allopolyploidy, and autoallopolyploidy, which is also a form of autopolyploidy.

### 3.3. Chromosomal Variations

Chromosomal variations are classified to two major categories in VariO [27]. Differences in the numbers of chromosomes are called aneuploidy. The other variant category includes those that change the chromosome structure. For detailed description and examples, see [27].

Aneuploidy is the most common cause of miscarriages and congenital birth defects. A total of 20% of human oocytes are estimated to be aneuploid [43]. Trisomy is an example of aneuploidy (Figure 1B). Down syndrome patients have a triplication of chromosome 21 [44]. The copy numbers of chromosomes vary in aneuploidy. In nullisomy, a certain chromosome is missing. The aneuploidy alterations range in size up to polysomy. Disomy, that is the normal state in many organisms, may still contain structural chromosomal variations. In heterodisomy, a pair of non-identical chromosomes is inherited from one parent, whereas in isodisomy a single chromosome has been duplicated from one parent. Uniparental disomy is a condition where both copies of a chromosome or a part of it are from one parent.

Numerous types of structural chromosomal alterations are known. Copy number variation, DNA mobile genetic element insertion, and nucleotide expansion are different forms of chromosomal amplification (Figure 1C). These variants can be interspersed, i.e., scattered in genome or be in tandem, when following each other. DNA transposons, insertion sequences, and retrotransposon insertions (LINE, LTR, and SINE segments, etc.) are mobile genetic elements that can move and duplicate within chromosomes. Nucleotide expansions have been classified as microsatellites (mononucleotide to hexanucleotide expansion) and minisatellites of up to few tens of nucleotides long repeats. Chromosomal deletions (Figure 1D) are either structural copy number variations, interstitial deletions in the middle, or terminal deletions in the end of the chromosome arm.

Chromosomal translocations are either intra- or inter-chromosomal (Figure 1E). A dicentric translocation is formed when two chromosome segments fuse together and when they both contain a centromere. In reciprocal chromosomal translocation, genetic material is switched between homologous chromosomes. The third type is Robertsonian translocation, where the long arms of two chromosomes are fused. The p arms are in this case very short in both the original chromosomes. In intrachromosomal translocation, a chromosomal region is translocated within a chromosome. Inversion is a special case where the translocated segment is located back to the original position but in reverse direction. If inversion appears within a chromosome arm, it is called paracentric. A pericentric inversion also contains the centromere.

Complex chromosomal variation is the outcome of at least four breakpoints (Figure 1F). Kataegis is a form of extremely complex chromosomal variation due to shattering of chromosome(s) into several pieces after which they are joined together. Immunological recognition molecule diversification of antibodies and B and T cell receptors (Figure 1G) is a complicated and special process that contains immunological receptor gene rearrangement, somatic hypermutation, gene conversion, and class switch recombination.

Isochromosome is a variation that lacks one arm while the other has been duplicated (Figure 1H). Ring chromosome has a circular structure (Figure 1I). Telomere length change is also a chromosomal structural variation (Figure 1J). It can mean either lengthening or shortening of chromosome ends, telomeres. Telomere healing means addition of a telomere to chromosome that has double-stranded break and deletion of a terminal segment.

### 3.4. DNA and RNA Chain Variations

DNA chain variations (Figure 1K) are local alterations and include DNA deletion, insertion, indel, substitution, and translocation [27]. These are among the most common variants within genes. Indels are variants that contain both an insertion and deletion [45]. Substitutions are either transitions between similar types of nucleotides (purine to purine or pyrimidine to pyrimidine), or transversion where a purine (A, G) is changed to a pyrimidine (C, T) or *vice versa*. DNA translocations also contain inversions.

Variations in RNA (Figure 1L,M) comprise RNA deletions, insertions, indels, substitutions, translocations including inversions, as well as missing RNA, and changes in initiation and termination codon [28]. In the coding regions, insertions, indels, and deletions are either in-frame or out-of-frame, indicating whether the amino acid code is retained or not. Missing protein is quite a common variant type. RNA quality control mechanisms, such as nonsense-mediated decay (NMD), destroy transcripts that contain premature stop codons due to substitutions, insertions, deletions, or indels.

Substitutions can be divided into several categories. Missense variants change the coded amino acid while synonymous variants do not affect the coded protein sequence. Nonsense variants introduce premature stop codons and such transcripts are often destroyed unless the variant is towards the end of the transcript, no more than 50 bp from the end of penultimate exon [46], or do not escape NMD due to various mechanisms [47]. Unsense variants [48] have largely been misclassified as synonymous, although they change the mRNA sequence due to regulatory alterations to splicing or expression regulation, or due to aberrant splicing. Thus, these variants are not synonymous, they often lead to mRNA degradation and missing RNA, thus causing missing protein, as well.

Figure 1M shows effects of variations on mRNA splicing. Transcripts can be alternatively initiated, terminated, or polyadenylated. These variants are common in many transcripts. Bases in mRNA can be modified. Alternative splicing is a very common phenomenon. Almost all human genes have alternatively spliced isoforms [49,50], but their biological significance is still largely elusive [51]. *Trans* spicing joins two different transcripts to form an RNA chimera.

*Cis* splicing within the transcript is the normal mode of splicing. Constitutively spliced exons are spliced in the order they appear in the mRNA. In exon skipping, splicing jumps over an exon. Mutually exclusive exons are a special form of exon skipping. In such a case, only one of a pair of exons appears in the final transcript. Loss of exon fragment means a case where a part of exon is spliced out. Inclusions can contain a fragment of intron or entire intron, called intron retention. Variation in the intron region can cause inclusion of a cryptic exon.

DNA variants can affect either exon, intron, or intergenic DNA. Cells contain organelles, some of which contain DNA. Organellar DNA variants include those of mitochondrial or plastid DNA. Other extrachromosomal variation forms are those in plasmids and extrachromosomal circular or linear DNA or RNA.

### 3.5. Protein Variations

Many variations in proteins are due to DNA and/or RNA alterations, therefore they are briefly introduced here, see Figure 1N. Amino acid substitutions, insertions, deletions, indels, and missing protein are the different forms of genetically caused amino acid changes [29]. Insertions and indels are either sequence retaining or amphigoric when the end of the sequence is coded by a frameshifted RNA. Deletions are either truncations or sequence retaining. Missing protein is a frequent consequence, e.g., because of a premature stop codon, mRNA out-of-frame alterations, aberrant splicing, etc. Single gene encoded proteoforms can be altered at DNA, RNA, and protein levels, see Section 2. Genes and genome.

### 3.6. Epigenetic Variations

Epigenetics means heritable changes that do not change the DNA sequence, and which affect gene expression and gene silencing. These changes can even be transferred by inter- or transgenerational epigenetic inheritance across several generations [52]. Epigenetic inheritance can also mean transmission of the epigenetic changes to daughter cells. Epigenetic markers may originate from internal regulatory processes or as responses to the environment of the organism.

**Figure 2:**
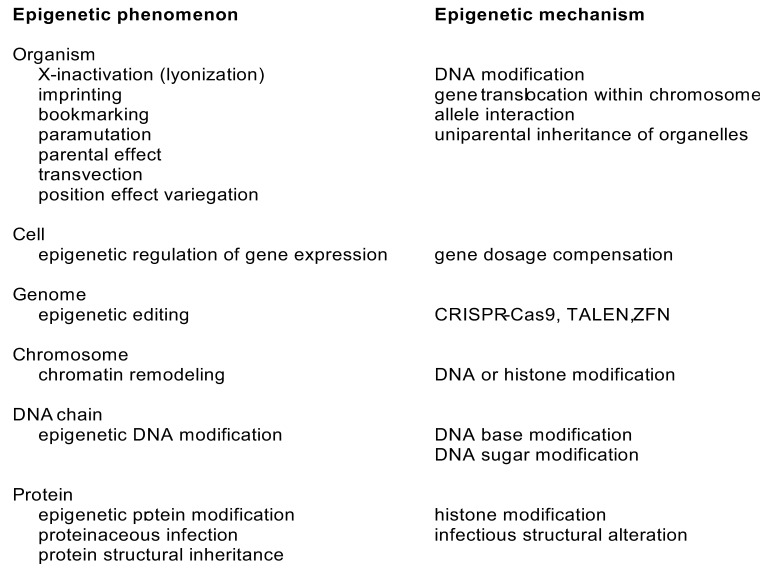
Novel systematics for epigenetic phenomena and processes. Data are presented at different levels from protein and DNA chain to organism level.

Epigenetic processes and mechanisms vary at different levels from DNA and protein up to organism (Figure 2). Epigenetics is based on modification of DNA or histone proteins in nucleosomes [53]. Although all types of DNA bases are modified in cells, epigenetic changes are known only in cytosine and adenine. They are incorporated by special enzymes: 5-methyl cytosine is the best-known example of epigenetic modifications (Figure 1O). It can be further enzymatically processed to 5-hydroxymethyl cytosine. Methylation of cytosines is especially common in CpG dinucleotides, but also in triplets of these bases. As many of these sequence segments have cytosine in both the DNA strands, methylations generate a strong signal. The methylation of adenine to N^6^-methyladenine is the other epigenetic alteration. In addition to these base changes, epigenetic alteration can occur also in the sugar moiety of DNA. Phosphorothioate modifications have been identified in bacterial restriction-modification system and may be more widely spread [54].

Protein post translational modifications are common and hundreds of different modifications are known. Histones are proteins that form the core in nucleosomes, protein-DNA complexes that facilitate further compression of DNA. A total of 145 to 147 nucleotides long stretch of double stranded DNA winds around a histone tetramer (Figure 1P). The formed nucleosomes appear like beads in a string. Charged and polar residues in the tails of the histone proteins are heavily modified (https://www.cellsignal.com/learn-and-support/reference-tables/histone-modification-table, accessed on 10 August 2022). The epigenetic histone modifications include acetylation, ADP-ribosylation, biotinylation, citrullination, methylation, phosphorylation, serotonylation, sumoylation, and ubiquitinylation. Changes in histones can affect chromatin remodeling and access of DNA with effects on transcription and further to gene expression [55].

Protein level epigenetic processes include protein structural inheritance and proteinaceous infection of prions [29]. Structural inheritance occurs in centrioles, the main microtubule organizing centers and regulators of cell cycle progression [56]. Asymmetric cell division or centrosome inheritance may be related to cancer [57]. Prion formation occurs in transmissible spongiform encephalopathies in humans and other species [58]. Prion proteins, which have undergone an irreversible structural conformational change, accumulate to amyloid fibrils in brains in the prion diseases. The transformed prion is insoluble and resistant to proteolytic degradation, and it also stimulates the conversion of normal proteins. The normal prion has functions and is abundant in the brain [59].

Chromatin remodeling means dynamic modifications to chromatin architecture. These changes affect access of condensed genomic DNA to the transcription machinery, and thus controls gene expression. Epigenome editing refers to targeted modification of epigenetic sites. Systems comprising clustered regulatory interspaced short palindromic repeat (CRISPR)-Cas9, transcription activator-like effector nuclease (TALEN), and zinc finger nuclease (ZFN) processes are known [60]. They appear naturally in some organisms and have been the target of intense research and development for medical and other applications, including gene therapy.

Gene dosage compensation occurs both at cell and organism levels. X-chromosome inactivation (XCI) and genomic imprinting regulate which alleles are active in cells. This is important for the compensation of X-chromosome numbers in females and generally for silencing one allele. XCI turns entire chromosomes off while genomic imprinting affects individual genes. Imprinting is maintained throughout the development of the individual or tissue. Human genome contains over 40 imprinted genomic regions [61] of both protein-coding and non-coding genes. Imprinting can lead to diseases if there is a genetic variation(s) in the expressed allele(s) [62]. Products of imprinted genes are involved in development, metabolism, growth, and behaviour. Imprinting relates to nine human diseases [63], including Prader–Willi syndrome due to deletions in the 15q11-13 region and consequent defective *SNRPN* and *NDN* gene products.

XCI, also called lyonization, silences randomly one of the X chromosomes in females and reduces expression level similar to males who have just one X-chromosome [64]. The dosage compensation of the additional X-chromosome in human females is virtually complete [65].

There are several additional epigenetic processes. Bookmarking means transmission of gene expression patterns during mitosis when gene transcription is silenced [66]. In paramutation, two alleles of a single locus interact leading to heritable change of one allele, induced by the other allele [67]. In transvection, an allele in a chromosome interacts with corresponding allele in the homologous locus in *trans* [68]. Structural (cortical) inheritance refers to transmission by spatial structures, such as the orientation of cilia in ciliates [69].

Parental effect is a process where, in addition to the genotype and environmental effects, also the environment and phenotype of the parent determines the phenotype of an individual. Maternal effects are due to mRNA or proteins supplied to the egg. Nucleolar dominance appears when ribosomal assembly sites, nucleoli, are inherited only from one parent or progenitor in the case of a hybrid [70].

Lightly packed DNA in euchromatin can be expressed, whereas condensed DNA in heterochromatin is silenced. A total of 56.1% of human chromatin is enriched in histone modifications [21]. Translocation or rearrangement of a gene within or near a heterochromatic region in a chromosome causes mosaic gene and protein expression patterns called position effect variegation. This is a common mechanism in plants but appears also in many other organisms. Genes within the heterochromatin are epigenetically silenced. An incontinentia pigmenti-like phenotype appears in patients whose Xq24-qter region is translocated to the 2q34 heterochromatin region [71]. The region contains a gene for the inhibitor of nuclear factor kappa B kinase subunit γ (*IKBKG*), expression of which is silenced by epigenetic DNA and histone alterations [71].

There is lots of variation in epigenetic marks. The changes differ between cells, and the marks are written and removed by active systems. Furthermore, genetic variations at the epigenetic modification sites and the genes for the activities for epigenetic changes can affect the epigenetic processes.

### 3.7. Selfish Genetic Elements

**Figure 3:**
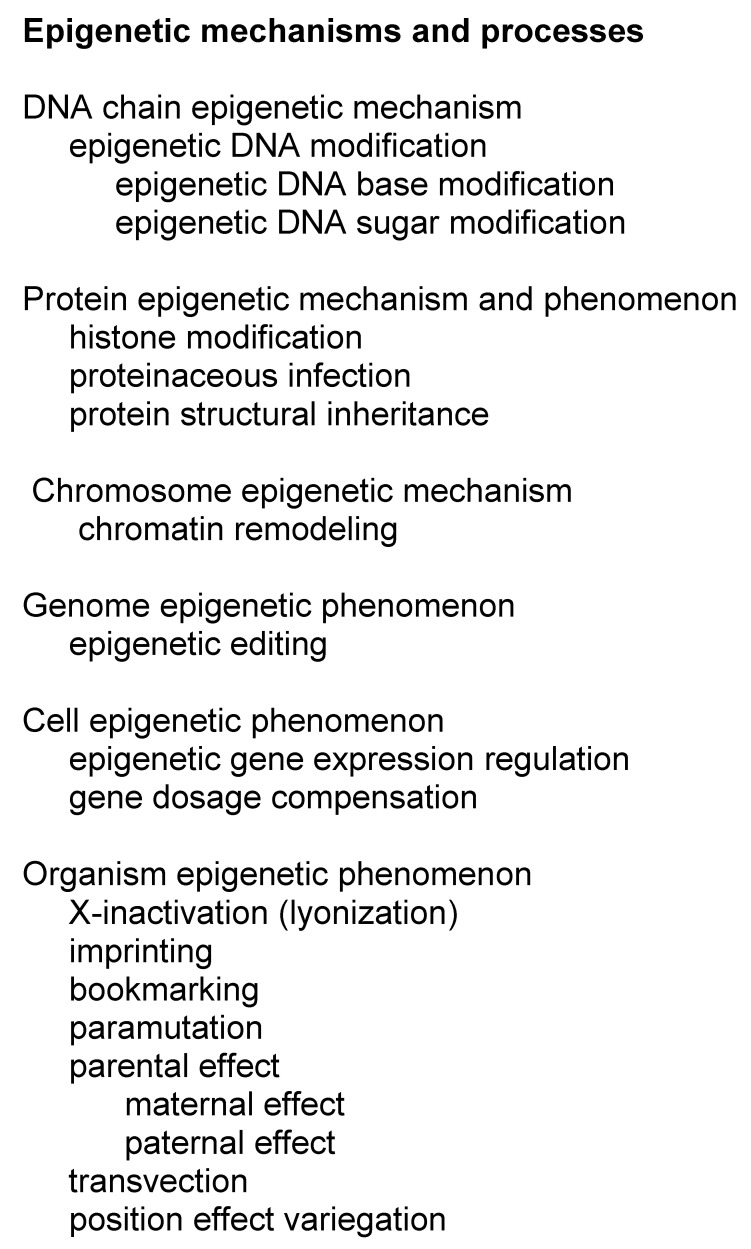
Novel systematics for types of genetic elements. The three major types are over-replicating elements, biased gene converters, and transmission distorters, each with more specific sublevels.

Selfish genetic elements enhance their number or transmission at the expense of other genes. This happens even when the action of a selfish genetic element has negative effect on the organismal fitness. There are three main types of selfish elements with subclasses (Figure 3).

First, over-replicating elements include mobile genetic elements and biased gene converters. Mobile genetic elements comprise transposable elements, plasmids, and endogenous viruses. Transposons are capable of multiplication of themselves by copying to different part of the genome. A total of 45.0% of human genome originates from transposons [72]. Second, homing endonucleases that insert preferentially into homologous uninserted sites are biased gene converters [73]. They belong to group I introns and code for nucleases that are self-splicing.

Third, transmission distorters affect the distribution of homologous chromosomes in gametes [74,75]. Meiotic drivers manipulate meiotic process and transmission of traits. Driving chromosomes and driving sex chromosomes affect segregation of selfish gene-containing autosomes and sex chromosomes, respectively. Similar mechanism also affects B chromosomes, which are common in many organisms. They are extra chromosomes and not essential for the survival of the individual.

Killer meiotic drivers are ultra-selfish elements that kill meiotic products that do not contain the DNA element. Gamete or spore killers are forms of killer meiotic drivers [76]. The killers have two main mechanisms, either killer-target system or poison (toxin)-antidote system [77].

The selfish gene in maternal effect dominant embryonic arrest (Medea) is composed of a toxin and an antidote in beetles [78]. A mother who carries *Medea* gene expresses the toxin in her germline and thereby kills her progeny. When also the progeny carries *Medea*, they produce the antidote and survive. When a mother has one *Medea* allele and one non-*Medea* allele, half of the offspring inherit the gene and survive.

Prokaryotic restriction-modification (RM) system can behave also as a selfish genetic element. RM systems are involved in, e.g., genetic transformation and infection prevention. Restriction endonuclease degrades DNA unless it is modified by the methylase. RM systems can act in certain situations as mobile genetic elements [79].

### 3.8. Genetic Variation in Microbiota

All higher organisms can be considered as symbionts as they constantly bear a very large number of microbes, archaea, bacteria, fungi, protists, and/or viruses. It has been estimated that the number of microorganisms in human microbiota outnumbers the human cells by about 1.3 times (Sender et al., 2016). The soma and the microbiota interact in complex ways, they affect each other, and even genetic conflict of resources may appear. The microbiota varies greatly between individuals and even within an individual in different situations and times. Microbiota and consequently microbiome vary during lifetime and even during seasons depending, e.g., on the available food, water, and environmental conditions. Microbiome is the collective genome of the microbiota in a system, such as microbiota of a multicellular organism.

Although there is heritable component in the constitution of gut microbiota, the environmental effect is more important [80]. We are only starting to understand the interactions between soma and microbiota and the significance, e.g., for the immunity, metabolism, health, and wellbeing of the organism [81,82]. The constitution of gut microbiota depends on diet, water, and other factors. It is possible to control microbiota to certain extent, e.g., with probiotics and food fibre [81]. Antibiotics and other drugs, pesticides, and other chemicals can have profound and long-lasting effects in the constitution of microbiota. The constitution of the microbiota may even contribute to parasite tolerance, as detected in *Caenorhabditis elegans* [83].

## 4. Mutation Rate

DNA alterations are frequent and comprise lesions, adducts, and structural variations. Several DNA repair mechanisms correct these errors and modifications, see TARAR countermeasures. Still some variants are inherited to daughter cells and to progeny. Lesion means an alteration to nucleic acid chain, and variation is an alteration that is transmitted further to daughter cells or offspring. The numbers of lesions are elusive and difficult to evaluate due to the very large number of variation types and mechanisms. The total number may well be over 100,000 lesions per day per cell [84,85], see Table 1. The number of abasic sites, one type of lesions, has been estimated to be about 30,000 every day in a mammalian cell [86,87]. The daily cellular frequencies of several other lesion types range from about 10 for double stranded breaks to 10,000 for single strand breaks [85], see Table 1.

Mutation rate means the frequency of new variants within certain time, such as during a generation or between cell divisions. It is highly variable at genomic regions, and depends on sequence context, dinucleotides, affected nucleotides, and of types of variations. Table 2 shows the mean numbers of human autosomal variation sites per genome for variation types based on data from about 2500 genomes representing 26 populations in the 1000 Genomes Project [88]. More than 99.9% of human variants are SNVs and short deletions or insertions. However, structural variants affect more bases. For 99.4% of the variants, the genotype is heterozygous, and the corresponding number for deletions and insertions is 99.0% [88].

The sperm mutation rate is substantially higher than that for eggs due to larger number of cell divisions. Human sperm cells have undergone approximately 659 cell divisions in a 40 year-old male, while oocytes have undergone just 31 cell divisions [84].

The average human germline mutation rate is about 1.2 × 10^−8^ per nucleotide per generation [89,90,91]. The human germline mutation rate is higher than for other investigated multicellular organisms [92]. However, when the number of cell divisions is taken into account, the human mutation rate is the lowest. The spectra of female and male variants are practically identical [89]. The human ratio of paternal to maternal variants is estimated as 3.5 [89].

Most studies on mutation rate have concentrated on SNVs. In humans, the combined rate of insertions and deletions has been estimated to be about 6% of that for substitutions [92]. The number of instances inversely correlates with the insertion or deletion length. There are frequencies also for other types of variants in Table 2.

Based on the SNV mutation rate, the number of *de novo* variants is estimated as 76.9 [89] or 64.4 [91]. The number of *de novo* variants increases with the age of father by 2.87 variants per year [89,93]. The rate of *de novo* short (≤20 bp) insertions and deletions is 2.94 and for longer than 20 bp alterations 0.16 variants per generation [94]. These variants affect on average 4.1 kbp of sequence and 29 bases in coding region per generation.

Mutation rates are significantly higher in unicellular organisms than in multicellular ones. Yeast *Saccharomyces cerevisiae* can grow either in a haploid or diploid form. The mutation rate is substantially higher in the haploid cells, 4.04 × 10^−10^ vs. 2.89 × 10^−10^ for SNVs, i.e., about 40% difference [95]. Ploidy did not affect the rates of deletions and insertions, 2.03 × 10^−11^ and 1.63 × 10^−11^, respectively.

Human somatic mutation rates vary for different types of cells. Germline cells have the lowest rates, while kidney tubules and appendiceal crypts have tens of times higher rate [96]. It has been estimated that at the age of over 60, for example, human intestinal epithelial cells that divide in every week or two have generated a variation in every position in the genome [92]. Comparison of somatic mutation rates in 16 mammalian species indicated large differences in rates per year; however, the lifespan somatic mutation burden did not vary more than 3-fold [97].

In addition to the genome, many organisms contain other genetic material. Endosymbiotic organelles contain DNA. The mutation rate of human mitochondria is about 3.6 times higher than germline rate, 4.33 × 10^−8^ per site per year [98]. Heteroplasmy, co-existence of different mitochondrial DNA (mtDNA) genotypes, appears likely in all individuals [99].

Several factors contribute to the mutation rate and include sequence context and the vulnerability of the site for variations, efficiency of DNA repair systems, fidelity of DNA replication, and chromatin structure [100,101,102,103].

The mutation rate of genomic regions varies widely; 100-fold differences have been reported [104], in the case of variants in 7-mers over 400-fold differences are seen in humans [105]. Y chromosome short tandem repeats (STRs) are widely used for paternity confirmation. The mutation rate of repetitive microsatellites ranges up to 7 × 10^−3^ per locus per gamete per generation in man [106]. Mutation rates in these segments vary over 700-fold, calculated from Fergus(s)on Y DNA Project data (http://dna.cfsna.net/HAP/Mutation-Rates.htm, accessed on 9 September 2022. Sequence context is an important factor [101], as there are mutation rate differences even in megabase sequence range [107].

Related to the sequence context, certain segments are highly vulnerable for variants. These include R loops, G quadruplets, and AT-rich segments [84,102,108]. Of dinucleotides, CpGs are 10-times more common among variations than any other dinucleotides [101,109]. Transitions are 1.7 times more common than transversions [92].

The CpG sites have been depleted from genomes during evolution. Instead of the estimated 4% in human genome, there are less than 2% present. Depending on the site, CpG transition rate can be several hundred-folds greater than for non-CpG transversions [110].

## 5. Variation Origin

Variants of genetic origin are grouped in VariO either as inherited, called germline variation, or somatic [16]. Somatic variants emerge in soma, cells of the body. These variants are due to numerous effects and mechanisms. DNA lesions appear in all cells that contain DNA and are largely repaired. However, some variants remain unrepaired and during the lifespan somatic variants accumulate [111,112,113]. Thus, the genomes in cells of an elderly person differ from the genomes the same individual had originally. There are also differences between cells and tissues.

Moreover, *de novo* variants either appear in one of the germ cells or early during embryogenesis. The variants are then inherited by all subsequent daughter cells.

Non-genetic origin means either artificial, edited, epigenetic, or modified variants [16]. Artificial variants are intentionally made. Genome editing with CRISPR-Cas9 system is an example of how edited variants are produced naturally in certain bacteria and archaea [114]. This mechanism is also widely used for artificial modifications. DNA methylation is the most common form of epigenetic DNA alteration [115]. Modified DNA is an outcome of, e.g., several types of lesions, such as chemical adducts.

### 5.1. Inherited Variants

Every individual inherits genetic material from their parents. This material has undergone processes that have generated unique variations. The chromosomes that the parents have inherited from their parents are stochastically segregated during meiosis when gametes are generated, thereby producing new combinations. During the first meiotic division, homologous chromosomes are independently assorted, and during the second meiotic division, non-identical sister chromatids are independently assorted. Gametes are haploid, i.e., contain just one copy of each chromosome.

**Figure 4:**
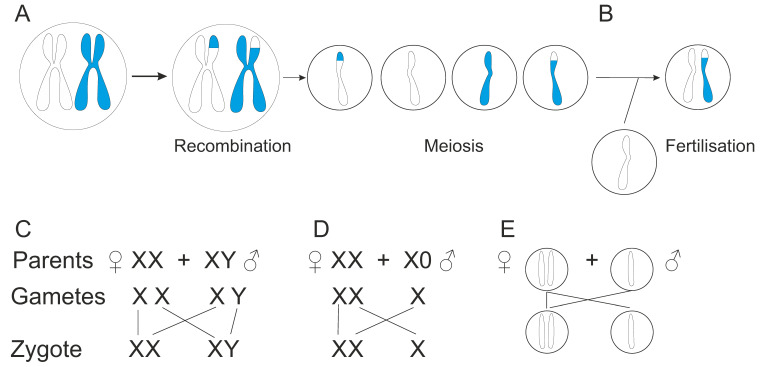
Sexual reproduction. (**A**) Generation of haploid sex chromosomes during meiosis. Genetic recombination between chromosomes can occur during the process. (**B**) The gametes are joined in fertilization. Sex determination of offspring (**C**) in XX/XY system. The homogametic sex can be also male, in ZW/ZZ system. (**D**) In XX/X0 system, only females have two sex chromosomes. The homogametic sex is male in ZZ/Z0 system. (**E**) Haplodiploidy, diploid females develop from fertilized eggs and haploid males from unfertilized eggs. For simplicity, only one pair of chromosomes is shown.

Further variations are produced during meiosis by genetic recombination by exchange of genetic material (Figure 4A). Homologous chromosomes form pairs in which DNA segments may be copied from one chromosome and attached to another without changing the donor chromosome. Another mechanism involves breaking and rejoining the DNA chains. Cross over between homologous chromatids leads to exchange of DNA segments. Genetic recombination can occur either during mitosis or meiosis. Mitotic recombination is rarer than meiotic recombination.

Homologous gene conversion is another mechanism that produces further variation by exchange of a homologous sequence between corresponding chromosomes or paralogous segments [116]. Gene conversions can be of three types: interallelic between alleles, or non-allelic (interallelic, ectopic) gene conversion, either in *cis* or *trans* [116]. Gene conversion appears predominantly during phases before mitosis, whereas mitotic crossing overs mainly take place during interphase. VDJ recombination is a special mechanism for joining genetic fragments to generate genes for immunological recognition molecules [20].

Recombination rates vary widely between genetic loci, populations, and organisms [117,118]. The autosome-wide average of recombination rate of Finns and non-Finnish Europeans were estimated to be 2.268 ± 0.4209 and 2.641 ± 0.5032 cM/Mb, respectively [119]. A 13 bp degenerate sequence motif is essential for recombination in large portion of the hotspot recombination sites and forms a binding site for PR/SET domain 9 (PRDM9), a histone-lysine N-methyltransferase [120,121].

Single cell sequencing to investigate meiotic variation in over 30,000 sperm cells from 20 young donors indicated rates from 0.010 to 0.046 aneuploidies per gamete [122]. There were from 22.2 to 28.17 crossing over events per cell.

Additional variation is produced by lesions at gametes or in the fertilized egg leading to *de novo* variants, variants that occur for the first time in a lineage. Cytoplasmic organelles are randomly distributed in mitotic segregation to daughter cells. Traditionally, organelles have been thought to be inherited in the egg.

### 5.2. Zygosity

Zygosity refers to different concepts. It is commonly used to describe similarity of alleles in a diploid system. Alleles are variants of a gene and can range in size from SNVs to large chromosomal rearrangements. In a homozygous organism, the alleles are similar, while in heterozygotes they differ. Compound heterozygous individual has two different alleles of a gene. When a diploid organism has only one copy of a gene, it is called hemizygous. Nullizygous organism has two loss-of-function null alleles or misses the gene completely.

Zygosity can refer also to allele origin. Autozygous alleles are due to non-random mating, whether due to inbreeding or consanguinity. They are called identical by descent. Identical by state is the name for allozygous alleles, which are due to random breeding.

Zygosity can also mean the genetic similarity of multiplets. For example, twins are either monozygotic or dizygotic whether due to single or double zygotes. In the case of monozygotic multiplets, the zygote has split to produce the siblings. Polyembryony is thus a form of clonal reproduction.

### 5.3. Somatic Genetic Heterogeneity

Human body consists on average of about 3.72 × 10^13^ cells [123], which are the outcome of about 10^16^ mitoses [124]. The accumulation of somatic variants has been assumed to be constant during life [96]. Most of these variants do not have effect or major phenotype, as they are just normal genetic variation and thus tolerated. Most forms of cancer are due to somatic variations, the number of which varies widely [4]. As the variation types have been discussed above, here the focus is on three somatic cell-specific phenomena: somatic mosaicism, microchimerism, and aging-related somatic variants. Somatic differences appear in all multicellular organisms; although most studies are for humans, there are data, e.g., for plants [125]. Human somatic variation profiles are tissue-specific and may have strand asymmetries [126].

Mosaicism is due to postzygotic variations that differ in somatic cells, while chimerism means that the individual is derived from two or more zygotes. In microchimerism, an individual has a smaller number of cells stemming from another individual. Hybrids are formed by breeding of two (sub)species, see Section 3. Types of Variations. Somatic hybrids are obtained when a nucleus is derived from one parent and cytoplasm is derived from both the parents, thus resulting in cytoplasmic hybrids, also called as cybrids. In heteroplasmy, there is more than one type of organellar genomes within a cell or individual. Mitochondrial cybrids have been used to study various diseases [127].

#### 5.3.1. Somatic Mosaicism

Somatic variations accumulate during the lifespan of all cells. Additional form of somatic variation is introduced in some organisms by programmed genome rearrangement [128]. The size of chromosome(s) is reduced (chromatin diminution) or some chromosomes are entirely deleted in somatic cells. Consequently, the genome is different in different types of cells within an individual. In the case of some ciliates, up to 95% of genome is deleted in somatic cells during embryogenesis and individual cells show heterogeneity [128]. Mosaic loss of Y chromosome (LOY) in men in leukocytes is the most common form of human clonal mosaicism, appearing in 2.5% at age 40 and 43.6% at the age of 70 [129].

As discussed above, somatic mutation rate is substantially higher than germline rate, varying according to cell type. The rate in kidney tubules and appendiceal crypts is about 50 times higher than in sperms and oocytes [96]. Despite the high-fidelity of DNA replication and DNA repair mechanisms, some new variants are introduced in every cell generation. Every single cell has thus somewhat different genotype, which is inherited by daughter cells, which again accumulate variations before dividing. Early variants of any size and type have large distribution and potential effect.

Variations in DNA repair system lead to accelerated aging and predisposes to cancers or both [130]. Variants that impair base excisions repair system are embryonic lethal [131].

Somatic mosaicism is related to certain diseases when genes are expressed outside their normal context. Cancer is an example of extreme somatic micromosaicism, and variants are different for each patient even for those having the same type of cancer. Certain cancers, such as lung cancer and melanoma, can contain even more than 1 million variations in comparison to other cells. Revertant mosaicism in a disease can restore partly or completely the wild type phenotype or activity by having reversion back to the original sequence [132].

Based on the distribution of mosaic cells, there are three categories: somatic, gonosomal, and germline mosaicism [133] which have been implicated in various diseases [133,134]. Most of the mosaicism remains unnoticed as there are not severe signs or symptoms. The clinical manifestations include, e.g., overgrowth or undergrowth, asymmetric growth, anomalies, streaky, or patchy pigment. Cutaneous mosaicism can cause striking patterns [133].

#### 5.3.2. Microchimerism

Various forms of chimerism have been classified [134,135]. Macrochimerism has been called also constitutional and classical chimerism and means that the cell lines from different zygotes are blended in an individual very early during embryogenesis. It is an extremely rare event. Microchimerism refers chimerism in smaller scale when there is a relatively small number of cells from another organism.

Microchimerism can be natural or artificial. Microchimerism due to medical procedures (e. g. blood transfusion or organ transplantation) is called iatrogenic. In orthotopic chimeras, the transplantation has been made to cognate, related position of the cells. When donor cells are transplanted in different location, heterotopic chimera is formed. Chimerism is essential for transplant tolerance [136].

Chimeras can be classified also based on temporal properties. In isochronic chimera, the time of the cells matches with the recipient, otherwise it is a heterochronic event.

Offspring obtain cells from their mother and have thus maternal microchimerism [137]. The cell transfer occurs also in the other direction so that mother has fetal microchimerism. Fetomaternal chimerism may persist for decades [138]. There are even XY cells when the female has had a male pregnancy. In a multipregnancy with non-monozygotic multiplets, the siblings share the placenta and obtain blood cell transfer from each other, and this is visible even when a sibling has aborted.

Gynandromorph is an organism with both male and female characteristics. They are chimeras that consist of genetically male and genetically female tissues. Gynandromorphs can be generated by several mechanisms: loss or damage of a sex chromosome, double fertilization of a binucleate egg, or symbionts [139]. Depending on the stage of development when gynandromorphism occurred, the individual may have bilateral asymmetry or mosaic distribution of the different cell types. This shows in sexually dimorphic organisms, such as butterflies and birds, as striking coloring patterns.

Chimera refers also to chimeric RNA transcripts that are formed by joining (parts) of two transcripts. This is a natural tissue-specific process [140].

#### 5.3.3. Ageing-Related Somatic Variation

Variations accumulate to cells during lifetime and are inherited to daughter cells. Progressive loss of physiological integrity along with impaired function are typical for aging. Out of the nine hallmarks for aging, three are directly related to DNA: genomic instability, telomere attrition, and epigenetic alterations [141].

It has been argued that DNA damage possibly affects all ageing phenotypes [142]. DNA repair defects cause several progeroid, i.e., premature aging, syndromes such as Werner and Néstor-Guillermo progeria syndromes, along with other symptoms [142,143]. DNA damages affect lesion correction, genome instability, mitochondrial function, metabolic alterations, epigenetics, telomere dysfunction, cell fate, etc. [85,113,142].

Clonal hematopoiesis is a common aging-related phenomenon in which mature leukocytes are derived from a single hematopoietic stem cell. Variations in this cell line are thereby enriched in the cell population and can contribute to diseases such as cancers and atherosclerotic cardiovascular disease [144,145]. Hematopoietic mosaic LOY is associated with cardiac fibrosis and heart failure [146].

## 6. Inheritance

Inheritance of genetic traits can be divided into two main categories: Mendelian and non-Mendelian inheritance. The former is called also monogenic inheritance due to being related to a single gene.

### 6.1. Monogenic Inheritance

Mendelian inheritance is named after Gregor Mendel who revealed the bases of inheritance: segregation, independent assortment and dominance. Autosomal inheritance refers to inheritance of traits genes for which are not located in sex chromosomes, i.e., in autosomes. Inheritance related to sex chromosomes is in humans and in many other species either X chromosomal or Y-chromosomal (holandric). Pseudoautosomal inheritance refers to inheritance of genes in pseudoautosomal regions of the X and Y chromosome that can exchange between the two sex chromosomes. Humans have 30 genes in this region [147]. Crossing over between X and Y chromosomes is usually restricted to these areas. Although located in sex chromosomes, the traits coded by these genes are inherited in autosomal fashion since both males and females have two copies of them.

Mendelian traits are either dominant or recessive in autosomes or sex chromosomes. In dominant diseases, one defective autosomal allele can cause an autosomal disorder. Huntington’s disease typically appears due to one defective copy of *HTT* gene for huntingtin that causes progressive degeneration of neurons in the brain. The number of CAG trinucleotide repeats in *HTT* gene in healthy individuals ranges from 10 to 26, while patients with the disease have extended repeat expansions [148]. Autosomal recessive trait is due to variants in both autosome alleles. Individuals with only one copy of the defective allele are carriers and mostly healthy. Cystic fibrosis (CF) is due to deleterious alterations in both alleles coding for the cystic fibrosis transmembrane conductance regulator (CFTR) [149]. CF affects the lungs, pancreas, liver, kidneys, and intestine.

Sex-limited traits appear only in one sex, although both sexes may have the allele that determines the trait. These genes are expressed in only one sex and therefore there is no penetrance in the other. This term refers to autosomal qualities only. Variants in *CELSR1* gene for cadherin EGF LAG seven-pass G-type receptor 1 causes hereditary lymphedema only in females [150].

Sex-linked inheritance is determined by a gene in a sex chromosome. *BTK* gene for Bruton tyrosine kinase is sex-linked as it is X-chromosomal. Boys with deleterious variations in this gene have X-linked agammaglobulinemia, a primary immunodeficiency, that blocks B cell maturation and leads to susceptibility for infections [151].

### 6.2. Non-Mendelian Inheritance

Inheritance that does not follow Mendelian rules is called non-Mendelian and consists of several different effects and mechanisms. They can be grouped into ten categories: non-Mendelian dominance, multigenic inheritance, tropopeissis, polypleyri interactions, gene dosage compensation, genomic imprinting, uniparental disomy, extranuclear inheritance, transgenerational RNA inheritance, and repetitive sequence inheritance. Non-Mendelian inheritance is equally important as Mendelian inheritance; the mechanisms are just more complex and there are numerous states.

#### 6.2.1. Non-Mendelian Dominance

According to the rules of Mendelian inheritance, traits are either recessive or dominant. Incomplete dominance and co-dominance are the two types of non-Mendelian dominance. The phenotype of a trait with incomplete dominance is intermediate. In codominance, both the different alleles of a locus are expressed.

Up to 16 genes may be responsible for human eye color and show incomplete dominance [152]. Human blood types describe red blood cells based on the presence or absence of inherited antigenic substances on their surface. ABO system describes blood groups A, B, and O. Alleles for the A and B blood groups are dominant. AB blood group bears the features of both the alleles due to codominance.

#### 6.2.2. Multigenic Inheritance

Multigenic inheritance, also called for quantitative or multifactorial inheritance, is common and in charge of numerous traits. Several genes contribute to these traits. Oligogenic inheritance is due to a few genes, whereas in polygenic inheritance a larger number of genes is involved. Many common diseases are due to multigenic inheritance together with, e.g., environmental factors [153]. These diseases include diabetes, cardiac diseases, cancer, and many others.

#### 6.2.3. Tropopeissis

Tropopeissis (τροποποίηση, Greek for modification) is a new term for gene interaction effects where genes jointly contribute to a phenotype. It is defined as an *interaction of genes or gene products that prevents partially or completely expression of the other gene.* It appears when one or more modifier genes mask, inhibit or suppress the expression of a gene. This phenomenon has traditionally been called epistasis; however, as there are different and contradictory definitions for epistasis [154], the phenomenon is here renamed tropopeissis to have an unambiguous term that can be used also in the VariO ontology. Epistasis has often been considered as a dichotomous phenomenon; however, the suppression may not be complete. The capabilities of mathematical and statistical models for epistasis to explain biological phenomena are limited [154]. Many multigenic traits are more complicated than interactions between two loci can explain.

Theoretically, there are 512 two-locus, two-allele, two-phenotype, fully penetrant disease models [155]. The number of nonredundant models can be reduced based on different assumptions between 50 and 102 models. When continuous penetrance values are considered, there are 387 distinct types of two-locus models [156]. By accounting symmetry between loci and alleles, the number can be reduced to 69. Another classification concluded there to be 33 shape symmetry classes [157]. There are many more interactions in systems that consist of more than two loci and where there is a range of effects. Here, some of the most common types of tropopeissis are discussed.

In dominant tropopeissis, a dominant allele at one locus can mask the expression of both alleles (dominant and recessive) at another locus. Recessive tropopeissis appears when recessive alleles at one locus mask the expression of both (dominant and recessive) alleles at another locus.

Dominant inhibitory tropopeissis means that a dominant allele at one locus masks the expression of both (dominant and recessive) alleles at a second locus. This is also known as inhibitory gene interaction. Duplicate recessive tropopeissis occurs when recessive alleles at either of the two interacting loci can mask the expression of dominant alleles at the two loci. In duplicate dominant tropopeissis, a dominant allele at either of two loci can mask the expression of recessive alleles at the two loci. Polymeric gene interaction describes a situation where two dominant alleles have similar effect when they are separate but produce enhanced effect together.

Awns are active photosynthetic structures in barley (*Hordeum vulgare*) and contribute to grain yield. Four pairs of genes are involved in awn development. Their interactions show different forms of tropopeissis interactions, reviewed in [158].

#### 6.2.4. Polypleyri Interactions

Multifaceted (in Greek polypleyri, Πολύπλευρη) one-to-many interactions occur when one gene influences several traits. Polypleyri interaction is defined as *a gene or its product(s) affects at least to some extent two or more phenotypes or traits.* The phenomenon has been called pleiotropy in the past, but similar to epistasis, the definitions of pleiotropy are fuzzy, confusing, and there is no consensus [159]. Therefore, an unambiguous polypleyri interaction is introduced instead. Polypleyri interactions describe interactions of genes and their products and do not directly describe fitness.

Polypleyri has several forms. See TARAR countermeasures for additional examples of polypleyri interaction mechanisms. For example, enzymes are promiscuous in two ways. Substrate and reaction promiscuity facilitate catalysis of various targets and processes, respectively [19]. Moonlighting activities are common additional functions that can be activated by variations [160]. A variant can have several different consequences even when affecting just one component. Albinism is an example of a polypleyri interaction mechanism. It is due to variants in a pigment producing enzyme. Tyrosinase (TYR) catalyzes the production of melanin and other pigments from tyrosine. The most severe form of albinism is called oculocutaneous albinism. People with this type of albinism have a wide spectrum of phenotypic characteristics including white or pink hair, skin, and iris color and vision problems [161].

#### 6.2.5. Gene Dosage Compensation

Diploid organisms have two copies of chromosomes, except for sex chromosomes in males. In organisms that have XY system, the Y chromosome is gene poor. Aneuploidies are largely harmful and detrimental; therefore, three different gene dosage mechanisms appear to correct for sex chromosome imbalance. Gene dosage compensation is important for balanced expression of proteins, e.g., in protein complexes. Differences in abundances of complex-forming proteins can be harmful [19].

In X-chromosome inactivation, one of the alleles in females is randomly silenced and it appears as tightly packed heterochromatin [162]. In humans, the dosage compensation is virtually complete in females [65]. In marsupials, only the paternal X-chromosome is inactivated [163]. XCI is based on epigenetic DNA modifications.

Another strategy to mitigate monosomy is upregulation of the X-chromosome in males. This process was initially detected in *Drosophila melanogaster* [164] and was subsequently detected in several mammals [165]. The third process is down regulation of X-chromosomal genes in females and has been noticed in *C. elegans* [166]. Condensin-like dosage compensation complex (DCC) binds to specific DNA sequence motifs and facilitates the regulation.

#### 6.2.6. Genomic Imprinting

Constitutive monoallelic expression of the same allele in the entire organism or tissue is called imprinting and originates from gamete DNA methylation, which is maintained throughout the development of the individual or tissue. There are over 40 imprinted genomic regions in man [61]. Genes in these regions and their products are involved in development, metabolism, growth, and behaviour. Variations in the expressed alleles can lead to diseases [62]; nine diseases are known [63]. Deletions in the 15q11-13 region cause Prader–Willi syndrome by paternal inheritance as *SNRPN* and *NDN* gene products are defective [167]. Maternal alleles are imprinted in Prader–Willi syndrome. Random imprinting causes somatic genetic mosaicism.

#### 6.2.7. Other Forms of Non-Mendelian Inheritance

Uniparental disomy can also be considered as a form of non-Mendelian inheritance since there is genetic material for a chromosome region just from one parent. The structural characteristics (the presence of two chromosomes) are maintained although the chromosome is from one parent. Uniparental disomy may remain unnoticed if there are no harmful variants in the duplicated chromosome. Paternal genome-wide uniparental disomy can cause Beckwith–Wiedemann syndrome when there is a harmful variant or variants [168].

Extranuclear inheritance refers to inheritance due to genetic material outside the standard chromosomes of the organism. Organelles, mitochondria and plastids (chloroplasts, chromoplasts, leucoplasts, and others), contain their own DNA and thus contribute to organellar inheritance. For example, mitochondria contain 13 protein, 22 tRNA and 2 rRNA-coding genes that are essential for the organelle function. Other proteins and, e.g., tRNA molecules are imported from cytoplasm. The distribution of variant containing organelles to sister cells leads either to homo- or heteroplasmy [169]. In homoplasmy, both the cells contain variant-containing organelles, while in heteroplasmy only one of the cells and its daughter cells contain both the alleles.

Mitochondria and mitochondrial DNA are predominantly maternally inherited and has been called strict maternal inheritance. There are also other forms of mitochondrial inheritance including paternal leakage of mtDNA, maternal inheritance of stable heteroplasmy, and doubly uniparental inheritance [170].

Doubly uniparental inheritance has been detected in various bivalve mollusks [171]. Mitochondrial DNA is typically inherited from the mother; however, in doubly uniparental inheritance, the situation is more complicated. Females inherit their mtDNA from the mother and transmit it to progeny of both sexes. Males inherit mtDNA from both parents, but they only transmit the DNA from their father. In female offspring, the maternal mtDNA is degraded whereas it is maintained in the males. In angiosperm plants, potential biparental plastid inheritance in chloroplasts is widely distributed [172].

Plasmids are common in many organisms and introduce plasmid inheritance [173]. Symbiotic infective particle inheritance (infectious heredity) refers to situation where the infectious particles, such as viruses, are transmitted to progeny [174].

Transgenerational RNA inheritance is typically contributed by small RNAs: microRNAs, small intervening RNAs, and piwi interacting RNAs [52]. However, the RNA types involved have not been extensively charted. The RNA molecules are involved in DNA methylation and histone modifications; there is not a clear picture on other effects and mechanisms.

Repetitive sequence inheritance is a special case of non-Mendelian inheritance. Certain short sequence stretches appear in variable numbers in genes and are linked to various diseases, more than 40 human diseases are known [175]. Repeats of three, four, and five nucleotides and even longer repeated segments are linked to diseases. These sequences can be expanded in successive generations in a non-Mendelian manner. This increase in severity or earlier occurrence of symptoms is called genetic anticipation [175].

## 7. Sexual Reproduction and Sex Determination

Reproduction of organisms can be either asexual or sexual. Parthenogenesis, asexual reproduction, is not reviewed here although some genetic exchange is possible in prokaryotes including transformation (acquisition of genetic material from environment), transduction (phage or bacteriophage-mediated gene transfer), and conjugation (direct unidirectional gene transfer between prokaryotic cells). The focus here is in sexual reproduction since it contributes significantly to the genetics and individual heterogeneity and has a number of benefits [176]. Sexual reproduction, the fusion of haploid gametes, is widely distributed; multicellular organisms that exclusively depend on asexual reproduction are very rare.

Gametes are reproductive cells produced in meiosis, reductional division, that generates cells with haploid genomes, i.e., containing just one copy of each chromosome pair (Figure 4A). In fertilization, haploid gametes fuse and form a zygote (Figure 4B). Thus, the new organism receives genetic material from both parents, which generates new genetic combinations and genetic diversity. Sexual reproduction produces genetically unique organisms. In endangered species and in inbred (in human called consanguineous) populations, the genetic pool is limited and leads to increase in genetic diseases and degeneration. The number of sexes varies, two is common, but there is wide range, *Schizophyllum commune*, a fungi, has > 20,000 sexes (mating types) [177]. Sexual reproduction generates variation that facilitates the survival of a species [176]. The variant combinations allow adaptation to changing conditions. Due to sexual reproduction, risk of diseases is reduced. Thereby, the evolutionary fitness may be increased. Sexual reproduction can help to select beneficial variations and decrease harmful variations.

There are also some disadvantages [176]. Sexual reproduction is energetically expensive and involves a long gestational period. It takes time and energy to find a mate and reproduce. Sexual reproduction is not a 100% successful method of creating offspring since some chosen mates may be infertile or incompatible. A favorable variant may not always be passed to the offspring, because of inheritance patterns. There are typically fewer offspring than in organisms that have asexual reproduction.

In organisms with binary system of sexes, there are males and females; however, variations to the binary sexes are common. Intersex individuals have either both types of sex organs or have sex characteristics of both sexes [178]. These individuals are often sterile. In human, about one out of 100 has some kind of differences in sex development [179]. Numerous organisms are hermaphrodites that have both types of reproductive organs and produce both types of gametes. In sequential hermaphroditism, the organism has one sex at a time. The other form is simultaneous hermaphroditism. Most flowering plants are hermaphroditic, there are also many hermaphroditic animals, mostly invertebrates. If hermaphrodites exist alongside males, the condition is called androdioecy and it appears, e.g., in corals and roundworms [180], while hermaphroditism alongside females is gynodioecy and is seen, e.g., in angiosperms [181]. In trioecy, there are in the same species males, females, and hermaphrodites. Some plants and animals have this sexual system [182]. There are even cases where male flowers and hermaphrodite flowers or female flowers and hermaphrodite flowers appear on the same plant. The situations are called andromonoecy and gynomonoecy, respectively.

Some organisms can undergo sex change from one sex to another. Sequential hermaphroditism is one form. Several fish species can change their sex [183].

The sex of an individual is determined during sex-determination process, which is a special case of inheritance. Sex determination is genetic in many species but can be also determined environmentally and called metagamic sex determination (Figure 4B). This can happen due to various reasons, including temperature or salinity change, photoperiod or crowding in the population [184], water pH, nutrition, body size, etc. Temperature-dependent sex determination has been investigated in reptiles [185]. Cytoplasmic and organellar sex determination occur in some species.

Sex determination can be related in different ways to fertilization. In progamic sex determination, the sex is determined before fertilization, such as in male honeybees. The sex is determined during fertilization in syngamic sex determination, see, e.g., [186]. Most plants and animals have this type of sex determination. If the sex is determined after fertilization, it is called epigamic. Female honeybees are an example, and the system is widely spread [184].

There are several genetic (also called genotypic) sex-determination systems, see, e.g., [187]. The following discussion relates to diploid organisms that have two sets of chromosomes and which is very common throughout taxon. XX/XY system (Figure 4C) is common in animals, including humans, but appears also, e.g., in some insects. X and Y are sex chromosomes, combinations of which determine the sex of the individual. Females have XX and males XY chromosomes. Some sex chromosome aneuploidies are known in human, such as X0 in Turner syndrome, XXY in Klinefelter syndrome, and trisomy X (XXX), XYY, and XXYY [188]. In XX/XY system the sex is determined either in X- or Y-centered way. Humans are an example of Y-centered sex determination, *SRY* gene for sex determining region Y in Y-chromosome defines whether the individual is male or not. In many insect, plant, fish, and mammal species, sex determination is polygenic [189].

The homogametic sex can be also male. ZW/ZZ system appears in birds, reptiles, and some insects [187]. In this case, females have the two different sex chromosomes, ZW, while males have ZZ chromosomes.

In XX/X0 system females have XX genotype and males have X0 genotype, which means that they only have one sex chromosome (Figure 4D). This system is quite common in insects. Algae have U and V sex chromosomes. They have two alternating phases in their life cycle. Gametophyte is the sexual phase where U and V are assorted to spores. V is for male and U for female.

In haplodiploidy, males develop from unfertilized eggs and are haploid, and females develop from fertilized eggs and are diploid (Figure 4E). This type of sex determination is common in insects. About 15% of arthropod species reproduce through haplodiploidy [190]. The sex of the haplodiploid organism is determined by the number of sets of chromosomes. Those formed from the union of a sperm and an egg develop as females. Interestingly, a male has no father and cannot have sons, but has a grandfather and can have grandsons. Recessive lethal and deleterious alleles are rapidly removed from such populations as the phenotype occurs always in males.

Paternal genome elimination appears in certain insects [191] and some other organism. It is quite similar to haplodiploidy but has a different mechanism. Males develop from fertilized eggs, but they pass to the next generation only the maternally inherited haplotype. Paternal genome elimination is a form of uniparental genome elimination.

In cytoplasmic sex determination, *Wolbachia*, an intracellular bacterial parasite, defines the sex in many arthropod species either due to killing or sterilizing males or by transforming them into phenotypic females [192]. The genotype of mitochondria defines the sex, e.g., in many gynodioecious plants [193].

## 8. Functions of DNA

DNA molecules have seven functions which have been systematized in VariO [16]. Information transfer is usually considered the most important, by some even the only DNA function. However, DNA has additional functions comprising regulation, transcription, DNA repair and replication, as well as reservoir of genetic material. Some DNA molecules have catalytic activity. The functions are discussed more in detail in [27].

Disease-causing variations are examples of alterations that change genetic information. Changes in 5′ region of a gene such as promoter and transcription factor binding site [194] or enhancer [195] affect transcription or DNA regulatory functions. DNA repair mechanisms are affected by several variation types and their context [196]. DNA replication fidelity is affected by many factors [197]. Some DNA molecules have catalytic deoxyribozyme activities and can catalyze sequence-specific DNA depurination [198]. Various genetic components can act as reservoir of genetic material [199,200].

The function of the gene is related to effects of variations in that gene. Essential genes are indispensable and harmful variants in them have effects [201]. Housekeeping genes [202] are common among essential genes; they are almost uniformly expressed in different cell types and tissues and independent of the condition.

## 9. Functional Effects of DNA Variations

Variants have a plethora of different consequences, effects, and phenotypes. Many genetic alterations are tolerated and thus have no effect on phenotype or fitness.

**Figure 5:**
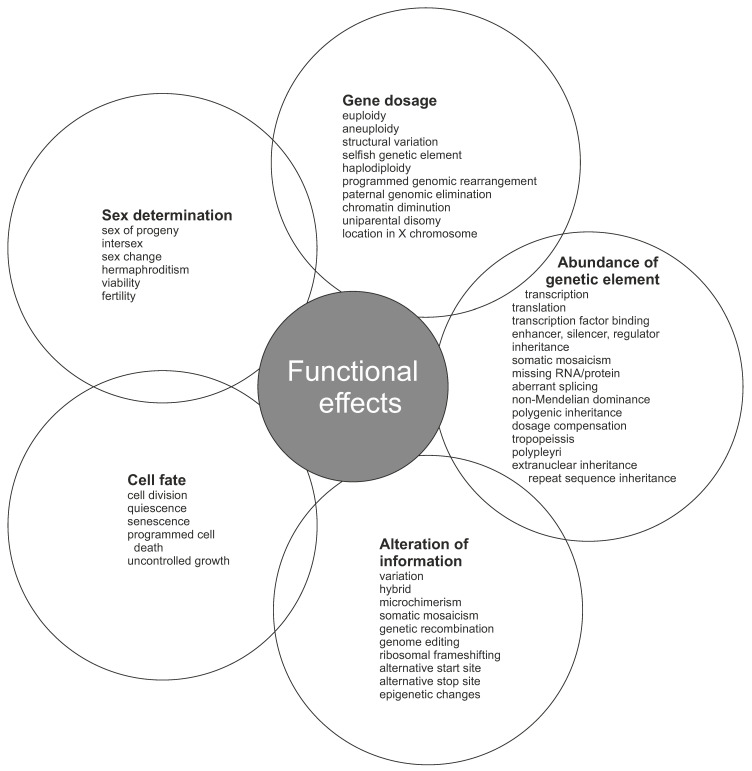
Functional effects of DNA. Gene dosage describes differences in the number of gene copies. Abundance of genetic element is due to factors in gene expression, regulation, dosage compensation, etc. Alteration of information class is for different variations and changes to the contents of genetic message. Effects to cell fate ranges from normal cell division to programmed cell death. In addition to the sex of the progeny and its possible change, sex determination category also contains effects on the progeny viability, fertility, and hermaphroditism. The indicated mechanisms may affect more than one functional category, visualized by overlaps of the shapes in the figure.

Functional effects of DNA variants are in the following organized into five categories (Figure 5). They are defined according to the same principles as the protein functional effects, including the biological systems, processes, and generalized consequences [19]. As the processes are described more in detail in other chapters, the mechanisms, effects, and processes are only briefly mentioned when organized into categories here.

Genetic variations either have no effect on the gene product, lead to gain-of-function (gof), loss-of-function (lof), and antimorphic or neomorphic effect. Neomorphic means novel activity while antimorphic activity is antagonistic.

### 9.1. Gene Dosage

Gene dosage has a deep impact on gene expression and gene product abundance. The abundance of genetic elements is affected by numerous mechanisms and processes, and it has fundamental effects on the usage of genetic information. Euploidy and aneuploidy describe the numeral changes in entire genome copies and chromosomes, respectively. Structural variations within and between chromosomes contribute to chromosomal amplifications and deletions with consequent alterations to gene dosage.

Selfish genetic elements aim to increase their number in a genome. A total of 45.0% of human genome consists of repeated selfish elements [72], but just less than 10 of them retain capacity to translocate [203]. In haplodiploidy, the ploidy of an individual depends on the sex of the individual, haploid for males and diploid for females. Programmed genomic rearrangements and (paternal) chromatin diminution reduce the number of genetic material and affect dosage of several genes.

Uniparental disomy reduces the number of different alleles to 1. Even location of a gene in a sex chromosome has a dosage effect. Harmful variants affect gene dosage also in somatic mosaicism. LOY causes mosaicism in leucocytes in men.

### 9.2. Abundance of Gene Products

There is some correlation between the gene dosage and the abundance of gene products; however, it depends on the gene. Gene expression is usually tightly regulated, and the abundance of gene products can thus vary widely. Housekeeping genes have rather ubiquitous, constitutive, and uniform expression in all normal cell types [202]. These genes code for central functions, e.g., in metabolism and signaling essential for all types of cells.

Variations that affect transcription, binding sites of transcription factors, promoter, start or stop sites, enhancers, silencers, and other regulatory segments all affect transcription and thereby gene product generation. Inheritance affects how many alleles are needed for a trait to show in the phenotype. Somatic mosaicism affects the abundance when there are different alleles and one of them, e.g., leads to mRNA degradation.

Missing RNA and missing protein are rather common outcomes of nucleic acid variants. Variants that impair the genetic code, such as stop codon-introducing variants at RNA level, out-of-frame insertions, deletions or indels, and variants that cause many types of aberrant splicing, are recognized by RNA quality control mechanisms and degraded, therefore no protein is produced. In certain situations, variants can escape from NMD.

Epigenetic changes affect gene expression either by modifying DNA or by affecting histone structure. The non-Mendelian dominance differs from the Mendelian dominance. Polygenic inheritance is due to complex interactions of genes. Dosage compensation mechanisms, XCI and genomic imprinting, silence genes, and chromosomes. Complicated genetic interactions, tropopeissis and polypleyri, along with extranuclear inheritance, transgenerational RNA inheritance, and repetitive sequence inheritance are other forms of non-Mendelian gene expression regulation.

The normal gene expression mode is biallelic. Still, many mammalian genes display random monoallelic expression (RME) [204], for details see [19]. They have mitotically stable allele-specific expression with different allelic states in clonal lineages. RME is widely spread and contributes to stochasticity in gene expression and heterogeneity in cells [205]. A total of 0.5% to 15% of autosomal human genes exhibit monoallelic expression, at least in some cell types [206].

### 9.3. Alteration of Information

Variants that change the genetic information, such as protein coding regions, can also change the carried information and affect RNA splicing, protein structure, function, or other properties. DNA lesions are very common—estimates reach up to hundreds of thousands per cell per day [207]. Despite efficient and high-fidelity mechanisms, some lesions escape repair and can become variations. Large body of the variations are well tolerated; they do not have a phenotype and do not affect the fitness of the individual carrying them, because there is no major alteration in the carried information. This is normal genetic variation, which is constantly generated, and which provides the foundation for biodiversity and evolution.

Hybrids are offspring of different species and thus contain different genome sets with somewhat different information. In microchimerism, the genes of another organism are in small minority; however, they still affect genetic information in the cells of foreign origin and in those interacting with them. Depending on the time when variants emerged, they are either inherited, *de novo* or somatic, and thus the spread of the potential alteration of information is either throughout the body or confined to a limited set of cells, tissues, or organs.

The numbers of somatic variations increase during time as they are inherited to daughter cells and lead to somatic mosaicism. Genetic recombination produces variations by cross-over and gene conversion. Genome editing modifies genetic information in specific locations. Ribosomal frameshifting leads to different types of transcripts and protein products. Differences are generated also by alternative start and stop codons. All the changes and effects listed above are caused by changes to nucleic acid sequences. Epigenetic changes can be inherited, but they do not change the sequence.

### 9.4. Cell Fate

DNA alterations also affect the fate of cells. Excessive number of variants within a cell leads to cell division arrest to allow the cell time to implement corrective processes. If not successful, cell becomes quiescent and cannot divide. Growth arrest is reversible in quiescence, while cellular senescence is non-reversible. In the most extreme case, programmed cell death clears cells that contain irreparable damage in their DNA. Uncontrolled growth leads to cancer.

### 9.5. Sex Determination

For sex determination of progeny, there are complex and organism-specific processes, which can be prone to DNA alterations. The consequences of such variants vary depending on the type of sex determination, whether genetic, environmental, cytoplasmic, or mitochondrial. Some factors can even cause sex change in certain organisms. Hermaphroditism is normal state in many organisms and enables sexual reproduction even despite of the original sex of individuals. Genetic conflict can affect both the viability (killing) and fertility of the offspring, especially males.

## 10. TARAR Countermeasures

**Figure 6:**
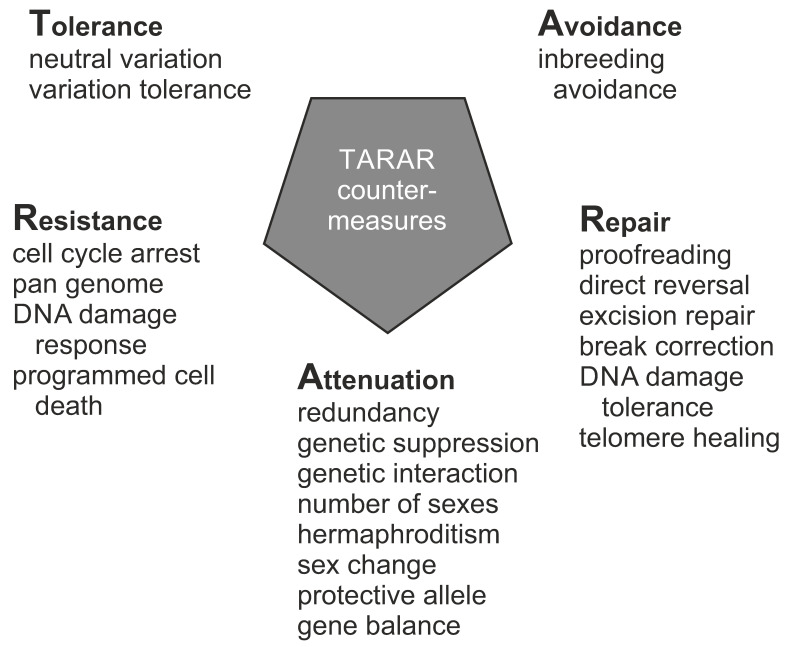
TARAR countermeasures for DNA variations.

Poikilosis in the form of genetic heterogeneity is pervasive and normal phenomenon and needed for evolution of species [5]. The extent of variation effects is restricted by TARAR countermeasures (Figure 6) that reduce the consequences of variations [5,208]. Various countermeasures reduce, restrict, tolerate, and repair effects of genetic variations. The relevant countermeasures depend on the type of variation, its location and effects, the cells where it is expressed, environmental, and other factors.

### 10.1. Tolerance

Many variants are tolerated and do not have a phenotypic effect. The ratio of neutral variants has been estimated and shown to vary largely depending on the gene and coded protein [209] and references therein. Analysis of all possible single nucleotide substitutions in all the 22 human mitochondrial tRNAs indicated that 49.0% of the variants are tolerated [210]. In short intervening RNAs (siRNAs), variants are more tolerated in the 5′ end than in other regions [211].

The protein coding part of eukaryotic genomes is relatively small; in the case of humans, the exons of protein coding genes cover 2.94% of the genome [21]. We are only starting to gain insight on the significance of the other parts of the genomes. Genomes are mainly transcribed but are apparently quite tolerant to many variations.

Variation tolerance in the coded proteins is largely context dependent and relates to the functional relevance of the affected positions in gene products. Catalytic sites are among the most conserved positions in enzymes. Even in these sites, some variations are allowed depending on the role of the protein [212]. Enzymes with the same activity are in this respect most conserved followed by enzymes catalyzing the same reaction for different substrates and those with different activities. Note that DNA damage tolerance mechanisms refer to repair, discussed below.

### 10.2. Avoidance

Many genetic alterations, such as lesions, cannot be avoided, as they appear stochastically at DNA sites; however, there are wide differences in mutation rates due to sequence vulnerabilities. Avoidance in genetics relates to the prevention of accumulation of potentially harmful variations due to inbreeding [213]. Inbreeding avoidance reduces risk of inbreeding depression: reduced fitness of inbred individuals. Mechanisms to avoid inbreeding include kin recognition, dispersal of individuals, preference for extra-pair/extra-group copulations, delayed sexual maturation of offspring, and reproductive suppression [213]. In certain species, females mate with several males to increase genetic diversity of offspring

Analysis of 41 species from six classes indicated that inbreeding avoidance is very general and depends on the risk of inbreeding depression in the species [214]. Some species have a preference for inbreeding; cichlid fish *Pelvicachromimis taeniatus* [215] and ground tit *Parus humilis* [216] are examples.

### 10.3. Repair

Repair mechanisms and processes correct and rescue DNA variants. DNA is vulnerable for a very large number of substances, physical effects and biological processes that produce, e.g., lesions and other changes. These changes can occur at any time and be due to internal or external factors. Alterations during DNA replication could be transferred to daughter cells. There are several DNA polymerases, depending on the organism, some of them have proofreading activity that substantially increases the fidelity of replication. For discussion on the fidelity of DNA polymerases, see [197]. Polymerases incorporate a wrong base approximately once every 10^4^ to 10^5^ nucleotides polymerized; however, the fidelity is increased by proofreading activities by 10^2^ to 10^3^ -fold. Many factors affect the fidelity: polymerase selectivity and proofreading, mismatch repair, a supply of nucleotides, and the type of error, and the sequence context [217]. The overall fidelity is one error per 10^8^ to 10^10^ nucleotides polymerized.

Direct reversal repair mechanisms are specific to the damage. Although there are many types of DNA alterations, there are only three dedicated reversal processes [218]. Photolyases reverse UV light-induced fusions of pyrimidine bases. O6-alkylguanine-DNA alkyltransferases reverse O-alkylated DNA damages. N-alkylated base adducts are reversed by AlkB family dioxygenases.

The double stranded nature of DNA facilitates many correcting measures. If the information is retained in one of the strands, it is used as the template for repairing the altered strand.

Single strand damages are corrected either by base excision repair (BER), nucleotide excision repair (NER), or mismatch repair [7]. BER systems correct modified bases and nucleotides first by cleaving with glycosylase, then AP endonuclease nicks the strand, after which DNA polymerase removes the apurinic or apyrimidinic site and adds a new base by using the other strand as the template. A larger stretch of DNA is repaired in NER. UV light induces formation of pyrimidine dimers between adjacent thymidines. These and other larger alterations are corrected by NER. Endonuclease removes the damaged region after which it is resynthesized with polymerase.

Mismatch repair system detects errors that have slipped through the polymerase proofreading activity. Depending on the organism, two or more proteins are involved in the detection and repair of these sites.

Double-strand breaks can be hazardous and lead to the deletion of substantial portion of a chromosome. Two different systems correct these variants [7]. In non-homologous end joining (NHEJ), a DNA ligase joins DNA strands. The process is often guided by short microhomologous segments in the ends of the strands. The process is not entirely accurate; it can introduce variations, as well as join segments that originally were not next to each other, not even in the same chromosome. Homologous recombination (HR) uses identical or nearly identical segment in a sister chromatid, or in a homologous chromosome.

DNA damage tolerance (DDT) pathways promote the bypass of single-stranded lesions during DNA replication [219]. The stalling of DNA replication fork can cause the fork to collapse and lead to DNA instability. In translesion synthesis (TLS), the DNA polymerase is temporarily replaced by a specialized TLS polymerase that can proceed through lesions. TLS is intrinsically error prone. In error-free template switching (TS), the stalled nascent strand switches from the damaged template to the undamaged newly synthesized sister strand for extension past the lesion.

Telomeres are special repetitive regions in the ends of chromosomes essential for replication [220]. They are shortened in every replication. Telomere healing means addition of telomere to chromosome parts which have been deleted and lost the telomere or did not previously have a telomere. Without the healing process, these chromosomes would not replicate.

### 10.4. Attenuation

Attenuation mechanisms reduce and decrease the consequences of genetic variations. Traditionally genetic buffering and genetic suppression are presented as attenuation mechanisms. Genetic suppression refers to intragenic and intergenic interactions that reduce the effect of a variant [221], whereas genetic buffering means various mechanisms and effects, such as modifier genes, gene interactions, environmental effects, phenotypic plasticity, and genetic redundancy [222,223]. These mechanisms act mainly at protein level, for discussion see [19]. Genetic redundancy functions at gene level and is thus discussed here.

Many genomes, including that of humans, have underwent one or more complete genome duplications during evolution. Thus, there have been lots of duplicated genes that have evolved further during time. Ancestral vertebrates had two rounds of whole genome duplication [224]. OHNOLOGS V2 database contains 7358 human two round ohnolog pairs [225]. Thus, many genomes contain redundant genes, products of which can attenuate effects of variants. However, even in yeast, a well studied model system, various estimates have been presented for the extent of buffering by duplicates, see [226].

One yeast study indicated that out of 201 duplicate gene pairs, 34% were at least partially redundant in a growth rate analysis [227]. A total of 49 cases of the redundant pairs (24%) were synthetically lethal, i.e., lethal when the variants appeared together. Analysis of totally eleven species, both prokaryotes and eukaryotes, indicated that the chance of survival due to gene deletion significantly increased when there was a duplicate gene [226]. The effects of duplicates are not fully understood. In yeast, genetic interactions of the duplicates were found to be only partly overlapping [228]. In addition, their expression patterns were not identical [229]. Many human disease-related genes have functionally redundant paralogs [230]. Moonlighting activities and promiscuity of proteins further increase the complexity [19].

Attenuation is related also to the essentiality of genes, as all genes are not indispensable and crucial for viability. Organisms can survive without many genes depending on the growth conditions. Several studies of human essential genes have each indicated ~2000 indispensable genes and proteins [231,232,233], although the gene sets overlap only partly. The essential proteins are mainly involved in developmental processes and sequence-specific DNA binding [234]. These genes are conserved, highly expressed, and they code for activities in DNA, RNA, and protein synthesis, many of which are abundant and involved in macromolecular complexes.

Risk or susceptibility alleles have been identified in relation to risk to certain diseases. Protective alleles attenuate and prevent disease phenotype. They are usually found from healthy individuals where their presence prevents disease, despite other disease-promoting (susceptibility) alleles at genes elsewhere in the genome. Protective alleles have been identified in various diseases [235]. Some of these alleles are evolutionarily old, for example, the genomic region that protects against severe form of COVID-19 was inherited from Neanderthals [236]; similarly, another region that is a risk factor for severe COVID-19 also originates from Neanderthals [237].

### 10.5. Resistance

Resistance countermeasures actively support and protect the genetic integrity. DNA damage response (DDR) triggers various cellular processes [238]. Signaling pathways related to ATM, ATR, and PRKDC are central for DDR [239]. The three protein kinases regulate both DNA repair and related processes that control cell cycle checkpoints and apoptosis.

Cell cycle arrest in multicellular organisms prevents the division of a cell when it contains a substantial number of variations and allows time for the repair measures. There are three types of growth arrest [240]. Quiescence is reversible, while cellular senescence is non-reversible and related to persistent stress due to, e.g., telomere attrition or oncogene activation. As the most extreme measure, the cell undergoes programmed cell death [241]. Bacteria have a related system called SOS response, which varies between species [242]. It involves cell cycle arrest and DNA repair. The third growth arrest type, terminal differentiation, is a permanent exit from the cell cycle.

Genetic resistance allows organisms to grow in stressful conditions. For example, in plants, genetic resistance suppresses or retards the development of a pathogen or an insect. Pangenome means the combined genes in an organism. A pangenome can include resistance providing genes; however, they may lead to reduced fertility, as in plants, seed production is reduced when a resistance gene is present [243].

Sex change or turn to hermaphroditism [183] in some organisms are examples of resistance when there either are not partners for sexual reproduction or when the balance of individuals with the different sexes is biased. These processes can be considered as types of genetic resistance counter mechanisms.

## 11. Intraindividual Genetic Conflict

The literature on genetic conflict has largely concentrated on the transmission of genes [244,245,246]. Intraindividual or intragenomic genetic conflict has been defined as a conflict between genetic elements when the phenotypic expression of genes promotes their own transmission [244]. However, the intraindividual genetic conflict manifests also as differential gene expression varying depending on genes. Fitness is relevant for intergenomic genetic conflict but is not covered here because it is population related.

Cells contain various types of genes which may have conflicts. Nuclear genes in eukaryotes are the major genes, but there are also cytoplasmic genes in mitochondria, chloroplasts, and other plastids, depending on the cell type and species. Genes can be classified as autosomal or sex chromosomal. In addition, there can be genes of mobile genetic elements, plasmids and intracellular microorganisms. Moreover, *de novo* variants can contribute to genetic conflict. Selfish action of a certain gene and its products changes the fairness of meiosis and the probability of traits to be transmitted to progeny. Genetic conflicts have various mechanisms.

Intragenomic conflicts have been classified into three categories based on origin, destination, and situation [245]. The origin conflict means conflict between autosomal and X-chromosomal genes in males, between sex chromosomal genes, and between cytoplasmic and nuclear genes. Autosomal genes are inherited from both parents, whereas X-chromosome in males is just from the mother. Genomic imprinting silences genes and causes differential expression for genes of maternal and paternal origin. Cytoplasmic genes are either in organelles, or in cytoplasmic pathogens or plasmids.

Destination conflict is due to the transmission distortion caused by selfish genetic elements [245]. There is even a conflict between autosomal and X-linked genes in males. The autosomal genes are transmitted equally likely both to male and female offspring, while X-chromosomal genes are transmitted only to female progeny.

Selfish genetic elements can have numerous effects on their host. These include increased mutation rate, shaping of genome and its function, evolution, fitness, fertility, viability, gene flow, formation of sex chromosomes, behavior, mate preference, and sex determination [75]. Both over replicating elements and transmission distorters (see 3.7 Selfish genetic elements) cause destination conflict. Homing endonucleases turn the affected alleles as homozygous. Medea is an example of lethal maternal effect by toxin-antidote system [78]. A mother who carries *Medea* gene expresses the toxin in her germline and thus kills her progeny. When offspring carry *Medea*, they produce antidote and survive. If the mother has one copy of *Medea* allele, one half of the offspring inherit the gene and therefore survive.

Cytoplasmic components, such as mitochondria, are typically inherited from the mother, which can lead to cytoplasmic genetic conflict. Organelles have various mechanisms to increase the production of female descendants. Human egg contains more than 100,000 mitochondria, while sperms have about 100 copies. Conflict can occur between the mitochondria from the parents. Paternal mitochondrial DNA is rapidly eliminated after fertilization. Some rare cases of biparental mitochondrial inheritance have been reported [247] and can be called as paternal leakage in the case of maternal inheritance.

Male-killing can be caused by inherited protists or bacteria, such as *Wolbachia*, in cytoplasm. Eggs from uninfected females can be inviable due to *Wolbachia*-containing sperms [248]. Another male affecting mechanism is feminization which converts males to females [249]. Male sterility is still another mechanism of cytoplasmic conflict [250].

The third type, situation conflict [245], of greenbeard genes [251] is related to intergenomic genetic conflict and therefore not discussed here.

## 12. Genetic Heterogeneity and Diseases

**Figure 7:**
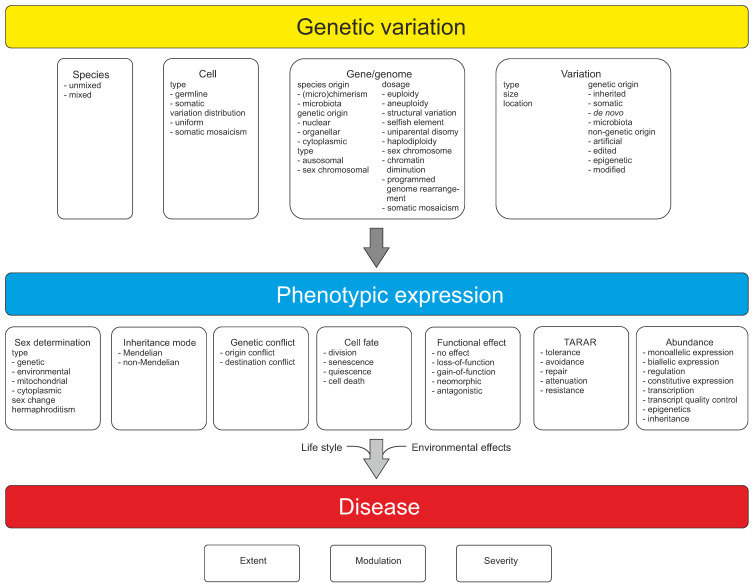
Genetic variation, phenotypic expressions and their contribution to diseases. Genetic variations affect phenotypic expression, which is further modified and affected by various factors and processes depending on the phenotype. Together with lifestyle and environmental factors, they contribute to diseases. The disease is described by its extent, modulation, and severity.

The medical interpretation of effects of genetic variation is most straightforward in single gene Mendelian diseases. The phenotypic expression of variants is affected by several processes, mechanisms and factors (Figure 7). Thus, despite having a harmful genetic alteration, the phenotype can be attenuated by TARAR countermeasures. On the other hand, e.g., genetic conflict could even increase the effect of a variation. In addition to the genetic component and phenotypic expression, lifestyle and environmental effects are pivotal in many conditions.

Any type and size of variation can cause disease. SNVs are the smallest genetic changes and large numbers of substitutions have been identified in thousands of diseases. Large part of variations, in many genes the majority, are benign and without phenotype. Experimental studies [252] and predictions [209,210,253] have indicated that genes display a wide range of vulnerabilities for genetic alterations. Even in Cancer Gene Census -classified cancer-related genes [254] with substitutions, just 40% of amino acid substitutions were predicted to be harmful [255]. Note that the results from massively parallel reporter assays, also called saturation mutagenesis studies, cannot be directly correlated to biological effect and disease relevance.

Genetic heterogeneity is pervasive in genetic diseases in many ways and levels. The inheritance pattern defines how inherited alleles interact. Figure 7 shows how genetic variation and introduced information alterations affect phenotypic expression and, once the effects are large enough, can lead to disease. The correlation between genetic variation and disease is most obvious in Mendelian diseases. Even in these cases, penetrance and phenotypes can vary even for individuals carrying exactly the same genetic alteration leading even to different diseases [256] and even in individuals having the same genetic background (monozygotic multiplets) [257,258,259]. Because of both genetic and phenotypic heterogeneity, it is necessary to increase sizes of cohorts in experimental investigations, e.g., in genome wide association studies [260].

Incomplete penetrance and heterogeneous expressivity appear almost in all diseases, at least to some degree. Variable expressivity and consequent heterogeneous severity of a disease are due to several factors and mechanisms, discussed in [261,262]. From the point of disease, genetic variation has many dimensions in addition to the nucleic acid changes, it depends also on which kind of cell the variation appears, what kind of distribution the variant has, and how the gene is expressed in those cells. Species origin as well as the origin and type of gene can modify the phenotype. Many types of variations alter the dosage of genes, chromosomes, and genomes and thus affect the abundance of gene products (Figure 5).

Genetic changes manifest phenotypic expression in multiple ways and levels. Sex determination is important for the individual as well as for the population. Inheritance mode defines how the genetic change is expressed. Genetic conflict and TARAR countermeasures modify and reduce the phenotypic expression, respectively. Severe genetic alterations are decisive for the cell fate. Functional effects and changes in the abundance of gene products modify the type and extent of activity change.

Diseases are continua and combinations of effects, symptoms, and mechanisms. Pathogenicity model (PM) describes diseases as the combination of three constituent factors: extent, modulation and severity, which are unique for each disease [263]. In the PM, the pathogenicity of a disease is described jointly by the distribution of the three factors in a cohort of healthy and diseased individuals. Extent measures the breadth of disease appearance. Severity of the disease indicates the state or degree to which the disease is expressed. Modulation means combined effects of many factors that modify disease phenotype, including some TARAR countermeasures, genetic conflict, and others.

Evidence-based cut-offs in the PM can be used for various purposes [263,264]. One such (curved) surface can be applied in diagnosis, another to define which cases are actionable, i.e., can be treated with existing regimes. The variability in the PM can explain also why some individuals benefit from a certain medication, while others do not respond or even have adverse drug reactions.

Somatic variations and those that emerge during development have diverse effects. Many of these variants are without phenotype, especially, if the gene with the variant is not expressed in cells that contain the variant. Variations accumulate during lifetime and contribute to somatic heterogeneity. The accumulation of genetic variations is one of the hallmarks of cancer [265] and important in many other diseases. In most cancers, majority of the variants are so called passengers that have little or no effect on the disease. Carcinogen-induced tumors, such as lung cancer and melanoma, can contain hundreds of thousands or more variations [4], most of which are passengers. Cancer is the most heterogenic and personal disease, since the genetic basis is different in every patient [3]. The accumulation of variants to key genes contributes to the progression of cancer.

The functional effects of genetic variants can vary widely even within a single gene disease. There are examples where the change depends on the position and/or type of a variant and has either gof or lof effect or no effect at all. Lof and gof classifications should always be based on experimental evidence [19]. In some genes, certain variants have gof effect while others are of lof type. In *PPP3CA* gene for protein phosphatase 3 catalytic subunit alpha, lof and gof variants lead to early onset epileptic encephalopathy and multiple congenital abnormalities, respectively [266].

Most current therapies of genetic diseases are targeted to the functional consequences of the genetic alterations. In gene therapy, the goal is to modify cells to generate therapeutic effect, often by correcting the defective gene. Hematopoietic stem cell transplantation is widely used to correct gene defects by introducing cells with fully functional versions of genes in primary immunodeficiencies, certain cancers, and some other diseases [267]. Recombinant viruses [268] and non-viral vectors (polymers, lipids, peptides, inorganic materials, hybrid systems) [269] have been used to transform cells by inserting genetic material to chromosomes. More recently, genome editing technologies have emerged and could allow direct correction of nonfunctional genes or gene products [270]. This is a form of curative treatment, but still in experimental phase.

Genetic counseling is an important part of genetic disease diagnosis and family planning [271]. The heterogeneity of the phenotypic expression and disease emergence make the task challenging. Consanguinity/inbreeding, which is common in some populations and species, influences the risk of inherited diseases.

## 13. Future Perspective

The gold standard methods for determining effects of variants are experimental, however, not possible to use to investigate all variants. In humans, 4.1–5.0 million sites differ in an individual in comparison to the reference genome, and about 10,000–12,000 of them cause amino acid substitutions [88]. Therefore, computational solutions are instrumental for the prediction of disease-relevance, called pathogenicity or tolerance, of various types of genetic changes [272].

There is place for improvement in variation interpretation. Although the performance has increased during years, still many use even decades old methods with poor performance [273]. NGS technologies generate genetic data sets and now even longer structural variations can be identified with high reliability, which has facilitated filling the difficult to sequence gaps in human genome and obtaining the complete sequence [72]. Problems emerge in the processing of identified variants. Widely used annotation tools provide some erroneous descriptions, especially for synonymous, splicing and truncation variants (Vihinen, submitted). Many disease-causing variants that are annotated as synonymous are nonsynonymous [48]. The consequences of RNA quality control [46] are largely ignored by the existing tools.

Most of the predictors for variation interpretation do not take the genetic and phenotypic heterogeneity into account; they have just two classes for benign and pathogenic variants. Only some methods consider the phenotype as a continuum. These tools include PON-P2 for amino acid substitutions in human [274], PON-All for amino acid alterations in all organisms [275] and PON-PS, a variant severity predictor for human amino acid substitutions [276]. Depending on the gene and disease, some portion of variants cause heterogeneous phenotype in individuals and cannot be classified in binary categories of benign and pathogenic. The International Society of Gastrointestinal Hereditary Tumours (InSiGHT) has classified variants in mismatch repair system genes for many years. They collect world-wide information for patients with a certain genotype and then apply the American College of Medical Genetics and Genomics and the Association for Molecular Pathology (ACMG/AMP) guidelines [277] and other criteria to classify the variants [278]. Currently, there are 6454 unique variants (data from InSiGHT databases in LOVD) for four genes (*MLH1*, *MSH2*, *MSH6*, *PSM2*). A total of 2453 (38%) of them are unclassified variants (UV) after several years of systematic data collection and classification. There will always be heterogeneity and some cases will thus remain in the UV class in all disease-related genes.

Variations have been collected and made available in numerous publicly available databases. These resources are instrumental for variation interpretation, clinical diagnosis, and research purposes. The funding of locus-specific variation databases (LSDBs) should be secured since they are typically the most reliable sources of variation information and curated by disease experts. LSDBs are too small to be considered and funded as infrastructures, and after initial publications, not eligible for research grants. Therefore, many of these valuable resources are maintained without dedicated funds, from the interest of the curators, a situation that is not sustainable for long. Large variation databases such as ClinVar [279], dbSNP [280], and dbVar [281] will continue as essential resources and have permanent funding. They should, however, pay more attention to record genetic heterogeneity and its phenotypic consequences.

The genetic heterogeneity and diversity have been largely ignored or considered as irrelevant in many studies. Wider ancestor distribution of cases is required, as the existing studies are often biased, dominated by individuals with European ancestry [282]. Variation interpretation in many conditions requires better consideration of lifestyle and environmental factors [283,284,285]. The inclusion of the effect of somatic variations in germline-based diseases and effects of microbiota are other important future research lines.

Our understanding is still very limited about the genetics of common, multigenic diseases. Rare monogenic diseases have been valuable in many ways in revealing genetic mechanisms. In common diseases, the interactions of genes and variants are relevant. In addition to coding genes, other types of functions have to be addressed more closely. For example, variants affecting regulatory functions need attention.

The investigations of variations and diseases need to step beyond the identification of genetic alterations. Functional studies to reveal mechanisms of the gene products [286] will, in addition to increasing understanding of variation consequences, phenotypic expression, and disease mechanisms, pinpoint novel drug and other therapeutic targets.

The contemplation of genetic and phenotypic heterogeneity is essential in population screening, prenatal testing, and newborn screening for improved detection rate. The availability of genomic sequencing is essential for implementing personalized/precision medicine [287,288]. Genetic heterogeneity is an essential factor and the complex interplay between numerous factors requires additional research and a paradigm shift from variation identification to variation effect understanding.

## Figures and Tables

**Table 1:** Numbers of types of DNA lesions per cell per day, data from [85].

DNA Lesion	Frequency per Cell per Day
Endogeneous	
Cytosine deamination	100
Cyclopurine adduct	100
Depyrimidation	100
Depurination	10,000
8-oxoG	1000
Monodialdehyde adducts	1000
Alkylation adducts	1000
Single strand breaks	10,000
Double strand breaks	10
Environmental	
Damaged bases	10
Photodimers	100
Single strand breaks	2–5
Double strand breaks	0.25

**Table 2:** Autosomal variant numbers per genome, median values. Data from [88]. The values indicate ranges in different populations.

Variation	Mean Value, Range
Variation type	
SNV	3.53–4.31 × 10^6^
Insertion or deletion	546,000–625,000
Large deletion	939–1100
CNV	153–170
Inversion	9–12
Mobile genetic element	1012–1222
Variation effect or site	
Non-synonymous	10,200–12,200
Synonymous	11,200–13,800
Untranslated region	30,000–37,200
Intron	1.68–2.06 × 10^6^
Transcription factor binding site	748–927
Promoter	81,600–102,000
Insulator	57,700–70,900
Enhancer	288,000–354,000

## Data Availability

Data are from the publications cited in each section. VariO has been updated to include the novel systematics presented in the text.
